# Definitive activation of endogenous antitumor immunity by repetitive cycles of cyclophosphamide with interspersed Toll-like receptor agonists

**DOI:** 10.18632/oncotarget.10190

**Published:** 2016-06-21

**Authors:** Soraya Zorro Manrique, Ana L. Dominguez, Noweeda Mirza, Christopher D. Spencer, Judy M. Bradley, James H. Finke, James J. Lee, Larry R. Pease, Sandra J. Gendler, Peter A. Cohen

**Affiliations:** ^1^ Department of Immunology, Mayo Clinic in Arizona, Scottsdale, AZ, USA; ^2^ Department of Immunology, Lerner Research Institute, Cleveland, OH, USA; ^3^ Department of Biochemistry and Molecular Biology, Mayo Clinic in Arizona, Scottsdale, AZ, USA; ^4^ Division of Pulmonary Medicine, Mayo Clinic in Arizona, Scottsdale, AZ, USA; ^5^ Division of Hematology/Oncology, Mayo Clinic in Arizona, Scottsdale, AZ, USA

**Keywords:** cancer immunotherapy, chemotherapy, TLR agonists, MDSCs, Tregs, Immunology and Microbiology Section, Immune response, Immunity

## Abstract

Many cancers both evoke and subvert endogenous anti-tumor immunity. However, immunosuppression can be therapeutically reversed in subsets of cancer patients by treatments such as checkpoint inhibitors or Toll-like receptor agonists (TLRa). Moreover, chemotherapy can leukodeplete immunosuppressive host elements, including myeloid-derived suppressor cells (MDSCs) and regulatory T-cells (Tregs). We hypothesized that chemotherapy-induced leukodepletion could be immunopotentiated by co-administering TLRa to emulate a life-threatening infection. Combining CpG (ODN 1826) or CpG+poly(I:C) with cyclophosphamide (CY) resulted in uniquely well-tolerated therapeutic synergy, permanently eradicating advanced mouse tumors including 4T1 (breast), Panc02 (pancreas) and CT26 (colorectal). Definitive treatment required endogenous CD8+ and CD4+ IFNγ-producing T-cells. Tumor-specific IFNγ-producing T-cells persisted during CY-induced leukopenia, whereas Tregs were progressively eliminated, especially intratumorally. Spleen-associated MDSCs were cyclically depleted by CY+TLRa treatment, with residual monocytic MDSCs requiring only continued exposure to CpG or CpG+IFNγ to effectively attack malignant cells while sparing non-transformed cells. Such tumor destruction occurred despite upregulated tumor expression of Programmed Death Ligand-1, but could be blocked by clodronate-loaded liposomes to deplete phagocytic cells or by nitric oxide synthase inhibitors. CY+TLRa also induced tumoricidal myeloid cells in naive mice, indicating that CY+TLRa's immunomodulatory impacts occurred in the complete absence of tumor-bearing, and that tumor-induced MDSCs were not an essential source of tumoricidal myeloid precursors. Repetitive CY+TLRa can therefore modulate endogenous immunity to eradicate advanced tumors without vaccinations or adoptive T-cell therapy. Human blood monocytes could be rendered similarly tumoricidal during *in vitro* activation with TLRa+IFNγ, underscoring the potential therapeutic relevance of these mouse tumor studies to cancer patients.

## INTRODUCTION

The treatment of cancer is currently being revolutionized by interventions which prove that the immune system can control not only early malignancies, but also advanced cancers. Successful immunotherapy can range in complexity from simultaneous administration of immune cells, cytokines and chemotherapy [1–5] to single agents, most notably monoclonal antibodies which block signaling of the Programmed death-1 (PD-1) receptor or its ligand, Programmed death-ligand 1 (PD-L1) [6–8]. Such PD-1 blockade effectively reverses inhibition of the natural effector T-cell response in up to nearly 60% of patients with melanoma as well as subsets of patients with many other cancers, resulting in major and often sustained therapeutic responses [6–8]. However, many patients’ cancers remain refractory to PD-1 blockade as well as to other current immunotherapy modalities; therefore, additional strategies are still needed for the majority of cancer patients.

On the most fundamental level, successful cellular immunotherapy requires accumulation and sustained activation of effector cells at literally all sites to which a cancer has spread. Cells of lymphocytic and/or myeloid lineage may serve as the final mediators of tumor rejection [9–17]. However, sustained effector activation is often or usually subverted by the strongly immunosuppressive environment typically generated by cancer throughout the body, recruiting regulatory T-cells (Tregs) and inducing myeloid-derived suppressor cells (MDSCs) as mediators of tumor escape [18–26].

Many chemotherapeutic agents have been reported to reduce host regulatory T-cells (Tregs) and/or MDSC subpopulations in addition to their direct anti-tumor effects [26–35]. Furthermore, the bone marrow rebound during each cycle of chemotherapy or whole body irradiation is associated with homeostatic, preferential proliferation of tumor-specific T-cells [33, 36–39]. However, even though each chemotherapy cycle may result in these favorable therapeutic effects, the benefits may be too transient to overcome the immunosuppressive impacts elicited by residual cancer. We hypothesized that a more sustained therapeutic benefit from chemotherapy could be achieved by administering repetitive cyclical chemotherapy along with interspersed Toll-like receptor agonists (TLRa). We predicted that this strategy could produce a substantial depletion of regulatory elements while causing the immune system to misidentify persistent tumor as a life-threatening infection, triggering a pronounced but appropriate escalation of both innate and acquired components of the endogenous anti-tumor immune response.

Existing literature supports the concept that coordinate administration of chemotherapy and TLRa can have greater therapeutic effects in mouse tumor models than either agent alone [40–44]. However, due to highly variable dosing, schedules and therapeutic impacts, it has remained unclear whether such therapy can be sustained to induce total and permanent eradication of advanced metastatic mouse tumors, and whether such a strategy can be successfully extended to human cancer.

In the course of our investigations in mice, it became apparent that the combination of chemotherapy and TLRa (the TLR9 agonist CpG-ODN 1826 (CpG) alone or with the TLR3 agonist polyI:C (pIC)) required 7 cycles of administration to consistently achieve its maximum therapeutic impact. This enabled identification of a well-tolerated treatment algorithm that can successfully eliminate a wide spectrum of advanced mouse tumors simply by coordinate repetitive administration of cyclophosphamide (CY) plus one or two TLRa. Surprisingly, even with repetitive chemotherapy, the success of the treatment was absolutely dependent upon the participation of T-cells already present in the tumor-bearing (TB) mice. Moreover, our studies suggested that endogenous T-cells served mainly a helper role, empowering CD11b+Gr1dim host myeloid cells to mediate tumor rejection in a nitric oxide (NO)-dependent manner.

## RESULTS

### Repetitive cyclophosphamide (CY) given with interspersed TLR agonist(s) (TLRa) eradicates a spectrum of advanced mouse tumor models

The three models we used for initial therapeutic screening contained a strong presence of regulatory T-cells (Tregs) [45–47], but ranged widely in MDSC (CD11b+Gr1+) content, from scant (Panc02) to moderate (CT26) to overwhelming (4T1) [25, 48–50]. 4T1 also displayed the most aggressive malignant behavior, abruptly metastasizing to multiple organs, and resulting in a tumor burden which has historically proved challenging to cure in wild type (WT) syngeneic mice [50–52] (Supplemental Figure S1).

We examined the therapeutic impacts of many variables, including different chemotherapy agents and TLRa, schedules and routes of administration, and lengths of treatment. We first determined maximum tolerated doses (MTD) when individual chemotherapeutic agents were administered repetitively to naïve mice on a weekly basis, approximating 21-28 day cycles in humans (Supplemental Figure S2A) [53, 54]. MTDs determined for naïve mice were, except for nab-paclitaxel, equally well tolerated in mice bearing advanced 4T1 or CT26 tumors (Supplemental Figure S2B and data not shown). Furthermore, TB mice tolerated treatment with TLRa alone (CpG ODN1826 and pIC) in a dose range and schedule that we had previously established as bioactive and well-tolerated (ref [55] and Supplemental Figure S2B-S2C). However, when chemotherapy agents were additionally paired with TLRa to treat advanced tumors, only CY+TLRa produced durable complete tumor regressions, a regimen which was remarkably well tolerated during seven weekly cycles of treatment (Supplemental Figure S2C). Other agents combined with TLRa were either well tolerated but ineffective (5-fluorouracil, irinotecan, sunitinib, and temozolomide) or poorly tolerated, obscuring any therapeutic efficacy (gemcitabine, docetaxel, paclitaxel, oxaliplatin, and doxorubicin. (Supplemental Figure S2C and data not shown).

Subsequent studies focusing on CY+TLRa revealed that substituting the TLR4 agonist LPS or recombinant IFNγ for CpG and/or pIC reduced therapeutic efficacy and was less well tolerated (data not shown). Intratumoral (i.t.) injection of CpG and/or pIC proved completely unnecessary for therapeutic efficacy, as remote intraperitoneal (i.p.) or s.c. TLRa also resulted in indistinguishable durable tumor eradication when interspersed with i.p. CY (Supplemental Figure S3 and data not shown). Remote (i.p.) administration of TLRa proved most effective and best tolerated when confined to only one dose at midcycle (day 3) (Supplemental Figure S3A), temporally corresponding to the onset of bone marrow recovery from CY-induced leukopenia. In addition, weekly CY doses < 2 mg (100 mg/kg) proved unreliable therapeutically, whereas 2-6 mg (100-300 mg/kg) dosing proved both effective and well-tolerated, paralleling human dosing of 300-900 mg/m^2^ (Supplemental Figure S3B and data not shown). In contrast, although daily CY dosing up to 0.8 mg (40 mg/kg) was well tolerated, tumor relapse was frequent at treatment's end (Supplemental Figure S3B), suggesting that daily CY's failure to induce cyclical rebounds from leukopenia was compromising to therapeutic efficacy.

Finally, a single TLRa (CpG given i.p. or s.c. midcycle) was sufficient for CY+TLRa to permanently eradicate a subset of advanced tumor models including CT26 and 4T1 (Supplemental Figure S3C and data not shown), whereas other models such as Panc02, KC (pancreatic), and C57mg (breast) only achieved transient rather than permanent regressions unless both CpG and pIC were administered (data not shown).

**Figure 1:**
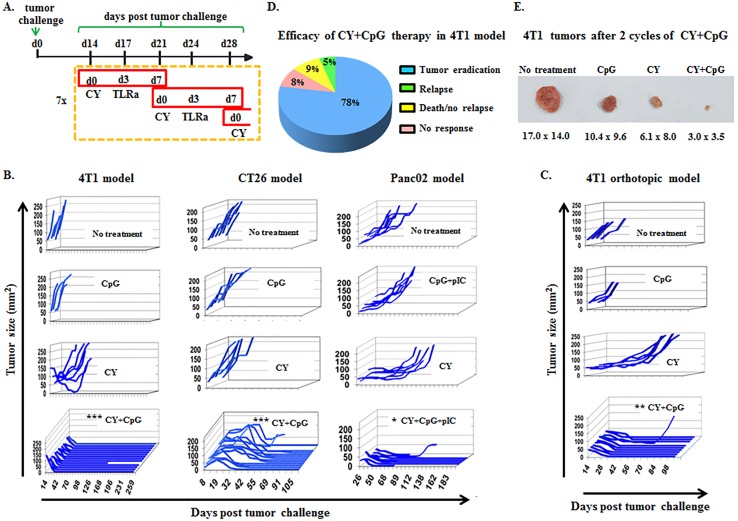
CY+TLRa treatment eradicates advanced tumors in multiple mouse cancer models **A.** Scheme of therapy protocol developed to treat 4T1 tumors. CY+TLRa treatment was initiated at d14 following tumor challenge, and was administered weekly for 7 cycles. Treatment schedule was similar for other tumor models, but instead initiated on d8 (CT26) or d36 (Panc02) of tumor-bearing. CY was administered at day 0, and TLRa was administered on day 3 of each cycle (solely CpG in the case of 4T1 and CT26, and CpG+pIC in the case of Panc02). **B.**-**C.** Tumor growth curves of untreated, CY-, TLRa-, or CY+TLRa-treated TB mice for individual tumor models. Each line represents a single mouse, plotted to show primary tumor size *vs* day post tumor challenge, showing the duration of survival. In the case of Panc02, mice receiving CY+CpG rather than CY+CpG+pIC initially displayed tumor regression in 6/7 mice, but only 1/7 mice achieved durable regression (data not shown). Individual mice were scored as complete tumor regression without relapse (eradicated) *vs* not eradicated, and analyzed by two-tailed Fisher's exact test. Statistical significance was determined by comparing CY+TLRa-treated group *vs* untreated, CY- and TLRa-treated groups for each model. **D.** Long term follow-up (150d) of > 100 CY+CpG-treated 4T1 TB mice. **E.** Representative 4T1 tumors from untreated, CpG-, CY- and CY+CpG-treated mice at cycle 2 day 3 (c2d3). Data shown in B and C are representative of ≥ 3 independent biological replicates for each tumor model. Larger numbers of mice received CY+TLRa to ensure robust survival end-points. Significance is shown as follows *p < 0.05, **p < 0.01, ***p < 0.001.

Combining these optimizations into a streamlined algorithm of 7 weekly cycles of i.p. CY (d0) and i.p. TLRa (d3) (Figure 1A), it was possible to achieve durable eradication of well-established s.c. challenges of syngeneic 4T1, CT26 and Panc02 tumors, as well as orthotopically implanted 4T1 (Figure 1B-1C). Although CY alone or TLRa alone variably delayed tumor progression, only the combination reproducibly resulted in durable complete responses, reflecting true therapeutic synergy (Figure 1B-1C). Long term follow-up revealed that mice treated with CY+CpG for 7 cycles achieved nearly 80% permanent eradication of macroscopic tumors in the 4T1 model (Figure 1D), with the bulk of macroscopic rejection already apparent by the end of cycle 2 (c2) (Figure 1E). Subanalysis of the orthotopic 4T1 model demonstrated 97% durable tumor eradication (data not shown). Permanent CT26 elimination was observed in up to 90% of challenges, and up to 55% of Panc02 challenges (data not shown).

In addition to the macroscopic tumors that were eradicated in the above studies, the pancreatic adenocarcinoma KC, the breast cancer C57mg and the sarcoma MC203 could also be durably eradicated by CY+TLRa. Two other models (B16 melanoma and MT breast) were not eliminated, but progression was attenuated for the entire period of therapy (data not shown).

### Efficacy of CY+TLRa treatment is T-cell dependent

**Figure 2:**
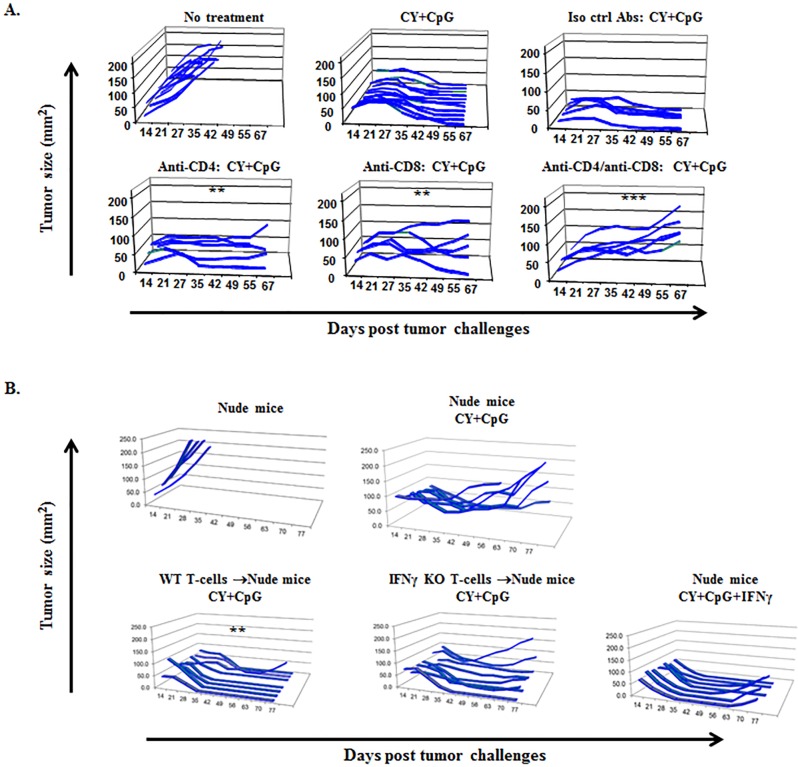
Endogenous T1-type CD4+ and CD8+ T-cells are required by CY+TLRa-treated hosts to induce sustained tumor rejection **A.** Tumor growth curves of WT 4T1 TB BALB/c mice variably depleted of CD4+ and/or CD8+ T-cell subsets, and treated with CY+CpG. Using two-tailed Fisher's exact test (durable tumor regression, yes or no), treatment without T-cell depletion was significantly different from groups receiving anti-CD4 (**p < 0.01), anti-CD8 (**p < 0.01) or both (***p < 0.001) depleting mAbs. Data in panel 2A are illustrative of 2 independent biological replicates in the 4T1 model (*n* = 5-12 mice per each condition in displayed experiment) and endogenous T-cell dependence was also observed in the CT26 and Panc02 models (data not shown). **B.** Athymic nude mice received either naïve WT T-cells, naïve IFNγ KO T-cells or none prior to 4T1 challenge. Subsequently, mice variously received treatment with CY+CpG or CY+CpG+IFNγ for 7 cycles. 4T1-TB nude mice treated with the CY+CpG regimen completely failed to reject tumor challenges in the absence of T-cell transfer. Compared to untreated mice, significant rescue of CY+TLRa's capacity to produce durable tumor rejections was observed with adoptive transfer of WT T-cells (**p < 0.01), but not with IFNγ KO T-cells (ns, *p* = 0.4667) or exogenous IFNγ (ns, *p* = 0.1923). Data in panel 2B demonstrating dependence of CY+TLRa treated nude mice upon adoptive receipt of IFNγ-producing splenocytes (not shown) or purified splenic T-cells (shown) are illustrative of 2 independent biological replicates in the 4T1 model (*n* = 5-8 mice per each condition in displayed experiment). Statistical comparisons in both 2A and 2B panels were performed using two-tailed Fisher's exact test. ns = non-significant.

Endogenous as well as adoptively transferred CD4+ and CD8+ syngeneic T-cells have been employed to induce rejection in multiple tumor models [15, 17, 56–60]. Given the fact that chemotherapy depletes effector T-cells as well as Tregs, we sought to determine whether the endogenous T-cell response was essential for CY+TLRa-mediated tumor rejection. We performed conventional *in vivo* T-cell depletions by administering anti-CD4 and/or anti-CD8 mAbs to TB mice (Figure 2A). These data showed unequivocally that enduring tumor rejection was dually dependent on endogenous CD4+ and CD8+ T-cells. Similar results were observed for Panc02 and CT26 tumors (data not shown).

To further investigate T-cell dependence, we used nude mice to control the properties of T-cells during the CY+TLRa therapy. T-cell-deficient nude mice on a syngeneic BALB/c background failed to permanently reject 4T1 challenges when treated with therapy that was fully effective for WT mice (Figure 2B upper panel). Transfer of unfractionated splenocytes or purified splenic T-cells from naïve syngeneic WT mice prior to tumor challenge enabled nude mice to respond fully to CY+TLRa, leading to sustained tumor eradication (Figure 2B lower panel and data not shown). To test the hypothesis that CY+TLRa tumor rejection depended upon a T1-type immune response, we transferred IFNγ KO rather than WT T-cells. The results demonstrated that CY+TLRa-mediated tumor rejection was strongly impaired in the absence of IFNγ-producing-T-cells (Figure 2B lower panel). Furthermore, even though repetitive administration of exogenous rmIFNγ with CY+TLRa enabled tumor rejection to occur in nude mice not receiving WT T-cells, such exogenous rmIFNγ could not replace the requirement for IFNγ-producing T-cells to achieve sustained tumor rejection (Figure 2B lower panel).

### Tumor-specific IFNγ-producing T-cells are evident in tumor-bearing mice

**Figure 3:**
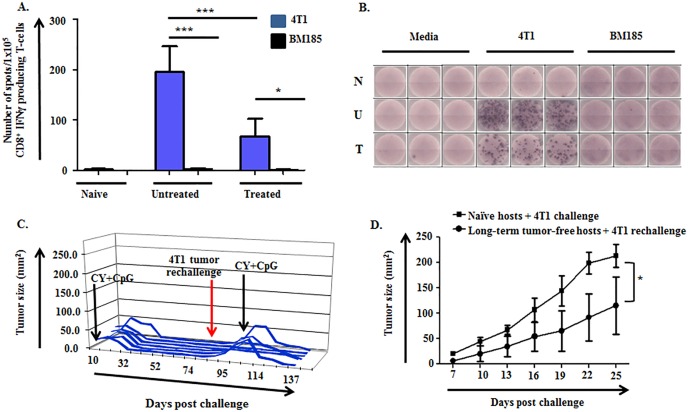
4T1-specific IFNγ-producing cells are induced in untreated and CY+TLRa-treated 4T1 TB mice, giving rise to immunological memory **A.**-**B.** Purified CD8+ T-cells from LN of naïve (non-TB), untreated TB, and CY+CpG-treated TB mice were evaluated for IFNγ production by ELISpot. Samples were studied at d28 of 4T1 tumor challenge, comparing untreated mice *vs* CY+CpG-treated mice at c2d3. A. Enriched CD8+ T-cells (1×10^5^ cells per well) were cultured in the presence or absence of irradiated 4T1 or BM185 tumor cell lines (1×10^5^ cells per well) for 48h. IFNγ production was analyzed as number of IFNγ spots per 1×10^5^ enriched CD8+ T-cells. Data are expressed as means ± SD, obtained from three independent biological experiments with three replicates per group. Statistical analysis was performed using one-way ANOVA with Tukey's posttest (*p < 0.05; **p < 0.01, ***p < 0.001). **B.** Representative ELISpot wells for naïve “N”, untreated 4T1 TB “U” and CY+CpG-treated 4T1 TB “T” mice. **C.** Long-term tumor-free CY+CpG-treated mice were rechallenged s.c. with 1×10^6^ 4T1 viable tumor cells. Mice displaying progression of the 4T1 rechallenge were again treated with CY+CpG for 7 cycles. Each line represents a single mouse (*n* = 6), plotted to show primary tumor size *vs* day post tumor challenge. **D.** 4T1 growth in challenged naïve mice *vs* rechallenged long-term tumor-free mice. Data correspond to 10 mice per group. Representative data from two independent biological experiments with similar results. Data are expressed as means ± SD. Statistical comparisons were performed using Student's *t*-Test.

Given the dependence of CY+TLRa treatment upon CD4+ and CD8+ T-cells as well as the requirement for IFNγ-producing T-cells for sustained tumor eradication (Figure 2A-2B), we inspected peripheral lymphoid organs for the presence of 4T1-specific T-cells. ELISpot analysis of spleen and lymph node (LN) demonstrated the significant expansion of 4T1-reactive IFNγ-producing-T-cells in untreated TB mice compared to naïve mice (Figure 3A-3B and not shown). Importantly, such T-cells persisted in the lymphoid organs of mice treated with CY+TLRa despite the latter's leukodepleting effects, but at significantly lower relative numbers compared to untreated TB mice (Figure 3A-3B) as well as lower absolute numbers (data not shown). In contrast, an absence of T-cell reactivity against another syngeneic tumor line, BM185, was observed in both untreated and CY+TLRa-treated 4T1-bearing mice.

The presence of tumor-specific, IFNγ-producing T-cells in CY+TLRa-treated mice prompted us to examine the development of immunological memory. When long-term tumor-free mice were subjected to second challenges (4T1, CT26 and Panc02), only 10-30% of mice promptly rejected the rechallenges (Figure 3C and data not shown). Importantly, mice with progressive rechallenges retained complete responsiveness to CY+TLRa retreatment, itself a T-cell dependent process (Figure 3C). Notably, even though only a minority of mice fully rejected rechallenges without CY+TLRa retreatment, immunological memory was evidenced by a significant delay in tumor outgrowth compared to similarly challenged naïve mice (Figure 3D).

### CY+CpG treatment differentially affects extratumoral and intratumoral microenvironments

**Figure 4:**
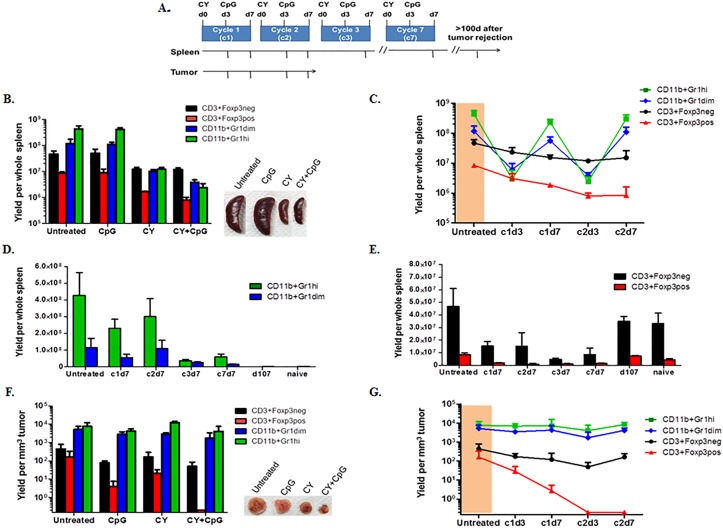
CY+TLRa repetitive treatment results in compartmental-dependent modulations of host myeloid cells, Tregs and effector T-cells **A.** Experimental design diagram; 4T1 TB mice were treated starting on d17 post-tumor challenge. When administered, CY was given on d0 and CpG on d3 of each cycle, for 7 cycles. At multiple time points (denoted by tick marks in the arrows), splenocytes and tumor-infiltrating leukocytes were isolated, enumerated, stained with mAbs against Gr1, CD11b, CD3, Foxp3, and CD45, and analyzed by flow cytometry to determine absolute and relative numbers of myeloid subsets (CD11b+Gr1hi and CD11b+Gr1dim), Tregs (CD3+Foxp3pos), and effector T-cell (CD3+Foxp3neg) subpopulations. **B.** Bar graph comparing the impacts of CpG, CY and CY+CpG *vs* no treatment upon splenic subpopulations in 4T1 TB mice at c2d3. Representative photos of spleens are also shown. **C.** Detailed dynamics of CY+CpG treatment upon splenocyte subpopulations during the first 2 cycles. 4T1 TB mice prior to treatment are highlighted in orange. **D.**-**E.** Comparison of intrasplenic CD11b+Gr1+ subpopulations **D.** and T-cell subsets **E.** from the beginning of CY+CpG treatment through to complete tumor rejection. **F.** Tumor-infiltrating leukocytes (CD45pos cells) isolated from 4T1 TB mice at c2d3 analyzed as in B. Representative photos of tumors are also shown. **G.** Detailed dynamics of the impact of CY+CpG treatment on intratumoral subpopulations analyzed as in C. Data are pooled from two independent experiments (*n* = 3-7 mice per time point). Data are expressed as mean ± SD. Statistical analysis presented in Results was performed using Student's *t*-Test. The data in panels B, C, F and G are presented in log10 scale. Statistical considerations are elaborated under Results.

We hypothesized that global cytoreductive impacts of CY+TLRa treatment depleted host effector T-cells (T_eff_), but to a lesser extent than MDSCs and/or Tregs, both extratumorally and intratumorally. Such a rebalancing might be sufficient for the endogenous T-cell response to maintain therapeutic efficacy, particularly if T-cells were not the final mediators of tumor rejection. To test this hypothesis, in the following set of experiments, CY was always given on d0 and TLRa (CpG) on d3 of each seven-day cycle to 4T1-bearing mice, and tumors were allowed to reach 10-12 mm diameter prior to commencement of therapy on day 17. Evaluations were performed at multiple time points (Figure 4A).

Because the most drastic reduction of tumor was observed during the second week (cycle 2) of complete therapy (CY+CpG) (Figure 1D), we performed comprehensive comparisons at cycle 2 day 3 (c2d3) of mice receiving either no treatment, CpG alone, CY alone, or CY+CpG. Analyses revealed that there were no significant differences detected between untreated and CpG-treated groups, whereas CY was mainly responsible for a significant depletion of intrasplenic CD11b+Gr1+ myeloid cells, both Gr1dim (monocytic) (**p = 0.0062) and Gr1hi (granulocytic) (***p = 0.0005) subsets, as well as significant reductions in CD3+Foxp3^neg^ T_eff_ cells (*p = 0.018) and CD3+Foxp3^pos^ Tregs (***p < 0.0001) (Figure 4B). Adding CpG to CY treatment resulted in significant further reductions of both CD11b+Gr1+ subsets (Gr1dim, ***p = 0.0006; Gr1hi, ***p = 0.0002) as well as Tregs (**p = 0.0016) without further reducing T_eff_ cells (ns *p* = 0.4335) (Figure 4B). However, whether derived from untreated or from heavily leukodepleted CY+CpG-treated mice, isolated splenic CD11b+Gr1dim cells were equivalently potent for inhibiting T-cell proliferation *in vitro*, verifying active MDSC function that was not apparent in the CD11b+Gr1hi fraction at this time point (Supplemental Figure S4). Therefore, CY or CY+CpG potently reduced the absolute numbers of intrasplenic CD11b+Gr1+ cells without impeding the inhibitory function of residual surviving CD11b+Gr1dim MDSCs.

We also examined more extensive time points for fully treated mice (CY+CpG) during the first two treatment cycles. Prior to any treatment, T_eff_ cells within the spleens of 4T1-bearing mice were outnumbered approximately 20-fold by total CD11b+Gr1+ cells (orange area, Figure 4C). However, as early as c1d3 of treatment, we observed preferential depletion of intrasplenic CD11b+Gr1dim and CD11b+Gr1high cells, leading T_eff_ cells to outnumber total CD11b+Gr1+ cells by approximately 6-fold. Nonetheless, between c1d3 and c1d7 there was a rapid intrasplenic re-accumulation of both CD11b+Gr1dim and CD11b+Gr1high cells, causing the latter myeloid elements in aggregate once again to outnumber T_eff_ cells 20-fold. A virtually identical depletion and rebound of intrasplenic myeloid cells was again observed during c2. In contrast, splenic Tregs were progressively depleted without the rebounds observed in myeloid cells. Reduction of T_eff_ cells was also observed without rebounds (Figure 4C).

We continued to analyze the spleens of CY+CpG-treated mice into the period when 4T1 tumors were no longer detectable (i.e., after cycle 3, c3d7) (Figure 4D-4E). The strikingly elevated accumulation of both CD11b+Gr1+ subsets observed in spleen pre-treatment and at the end of cycles 1 and 2 (c1d7, c2d7), became less pronounced with subsequent cycles (c3d7-c7d7). Nonetheless, mildly elevated levels of intrasplenic CD11b+Gr1+ cells persisted as long as the treatment continued, and several additional weeks were required after cessation of weekly CY+CpG for spleens to regain the same cellular proportions of myeloid and T-cells as those observed in naïve spleens (Figure 4D-4E). Parallel analyses of lymph nodes showed trends similar to those observed in spleen (data not shown).

In contrast to our analyses in spleen and lymph nodes, studies of intratumoral (4T1) constituents at c2d3 of treatment (Figure 4F) revealed that T_eff_ cells proportionately remained vastly outnumbered by CD11b+Gr1+ myeloid cells regardless of whether they had been exposed *in vivo* to CpG, to CY or to both. This finding raised the possibility that, in comparison to spleen, both intratumoral CD11b+Gr1+ subsets displayed CY resistance. Nonetheless, mice receiving CY+CpG were concurrently engaged in successful tumor rejection (Figure 4F). Furthermore, dual CY+CpG treatment was uniquely associated with a progressive, virtually total elimination of intratumoral Tregs when compared to the other groups (*vs* untreated *p = 0.0454; *vs* CpG *p = 0.0321; and *vs* CY **p = 0.0085) (Figure 4F), suggesting reversal of immunosuppression in the tumor microenvironment. The absence of cyclical fluctuations of intratumoral myeloid subsets was also observed during the more extensive time point analysis of CY+CpG-treated mice (Figure 4G). In addition, durable depletion of Tregs was found to persist throughout the period of tumor rejection, accompanied by a modest progressive accumulation of intratumoral T_eff_ cells.

Since myeloid cells constituted the major surviving subpopulation within CY+CpG-treated, regressing 4T1 tumors, we investigated the possibility that this treatment radically shifted the function of CD11b+Gr1+ cells from immunosuppressive to tumoricidal.

### Tumoricidal function is activated by CY+TLRa treatment

**Figure 5:**
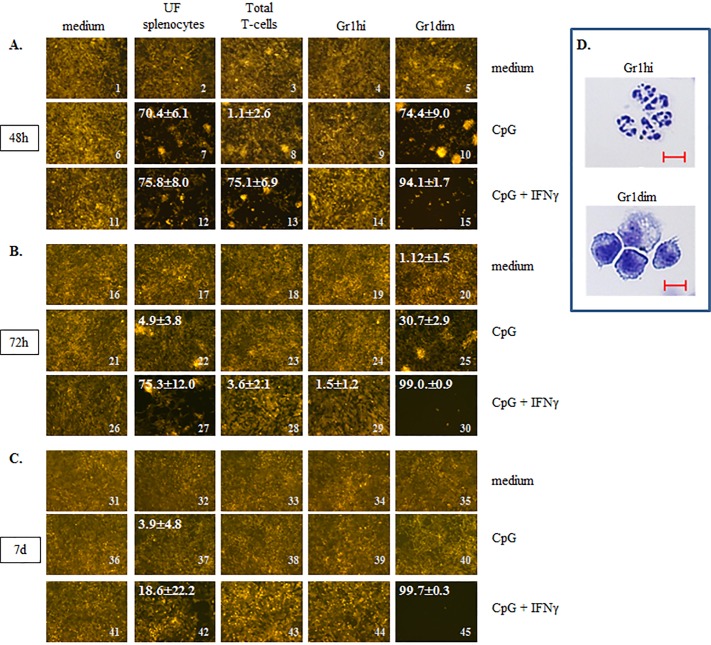
CY+CpG treatment induces unique tumoricidal properties in myeloid Gr1dim cells **A.**-**C.** Evaluation of induced tumoricidal properties by CY+CpG treatment. Unfractionated splenocytes “UF”, total T-cells, and myeloid Gr1+ (Gr1hi and Gr1dim) cells from spleens of CY+CpG-treated 4T1 TB mice were co-cultured with non-irradiated 4T1-f cells in the presence or absence of CpG alone (1 μM) or with IFNγ (1000 U/ml), and tumoricidal activity (acellular black areas) was evaluated at 48h, 72h and 7d in culture. The percentage of tumor growth inhibition, denoted in white numbers (top left), was calculated as the average of percentages from 6 non-overlapping fields ± SD. Images without percentages represent confluent monolayers (killing below detection levels). Images were numerically labeled on the bottom right for the purpose of identification in the Results text. The *in vitro* assays were performed with 2×10^4^ non-irradiated 4T1-f cells and 1×10^5^ leukocytes added to the assays at time 0, and representative photos are shown. Photos were taken using a Zeiss Axio Observer A1 microscope. **D.** Representative photos of isolated Gr1hi and Gr1dim cells from spleens of CY+CpG-treated mice. Scale bar, 10 μm. For all the assays, 10-12 CY+CpG-treated mice were pooled per experiment at c2d3. Data are representative of three independent experiments run in duplicates or triplicates with similar results. Statistical analysis presented in results was performed using Student's *t*-Test.

To determine whether weekly CY+TLRa treatment induced tumoricidal properties in leukocytes of CY+TLRa-treated mice, we evaluated the killer capacities of splenic leukocytes isolated from CY+TLRa-treated and from untreated 4T1-bearing mice (Figure 5 and Supplemental Figure S5). We compared unfractionated splenocytes (UF) to isolated CD11b+Gr1hi and isolated CD11b+Gr1dim fractions. In addition, we evaluated isolated splenic T-cells because of the dependence of tumor eradication upon the endogenous T-cell response (see Figure 2). Finally, given the fact that endogenous T-cell-derived IFNγ and exogenous TLRa were both essential for tumor rejection in CY+TLRa-treated mice (Figure 2B), we also examined the capacity of added IFNγ and/or CpG to modulate host leukocytes’ tumoricidal function *in vitro*.

To assess both the short-term and long-term impacts of host immune leukocytes upon unbridled cancer growth, we devised an extended 7-day assay in which non-irradiated tumor cells stably expressing fluorescent protein were used as targets. For example, in the case of 4T1 assays, fluorescent 4T1 tumor cells (4T1-f) were plated at 20,000 cells per microwell (time 0), a starting concentration which in medium alone enabled 4T1-f to proliferate to confluence within 48h, yet still maintain high viability during subsequent overgrowth out to day 7 of culture (Figure 5A-5C, first column). CpG alone or with IFNγ was also variably added to microwells at time 0, and we observed that these factors by themselves did not detectably impact tumor proliferation or survival (Figure 5A-5C, first column; for impacts of IFNγ alone see supplemental Figure S5). Also at time 0, unfractionated (UF) splenocytes or isolated splenic subpopulations obtained from CY+TLRa-treated or untreated 4T1-bearing mice, were variously added at 100,000 cells per well (Figure 5 and Supplemental Figure S5). Viable tumor cells were assessed under fluorescence and bright field at 48h, 72h and 7d after plating, and percentage of tumor cell inhibition (black areas) relative to wells not receiving host cells was quantified with ImageJ software, based on the evaluation of 6 non-overlapping high power (400x equivalent) fields (HPF) per microwell. Each condition was furthermore set up in 2-3 separate microwells to verify technical reproducibility, and each condition was run from scratch at least twice to assess biologic reproducibility of observed trends.

Our analyses of splenic leukocytes showed that freshly isolated, UF splenocytes from CY+TLRa-treated TB mice had no impact upon 4T1-f growth in culture in the absence of added factors at any evaluated time point (Figure 5, images 2, 17 and 32). In contrast, in the presence of CpG+IFNγ, UF splenocytes significantly inhibited 4T1-f growth at 48h and 72h (Figure 5, images 11 *vs* 12 and 26 *vs* 27 ***p < 0.0001 in both cases); however, this impact was markedly diminished beyond 72h (Figure 5, image 27 *vs* 42 ***p < 0.001). A less sustained effect was observed when UF splenocytes were exposed to CpG without IFNγ (Figure 5, images 7, 22 and 37). Similarly, freshly purified total T-cells from the spleens of CY+TLRa-treated mice also showed an absence of anti-tumor impact *in vitro* unless CpG+IFNγ were also added to culture (Figure 5, image 11 *vs* 13 ***p < 0.0001). Again, this was a transient effect which was not sustained beyond 48h (Figure 5, image 13 *vs* 28 ***p < 0.0001).

When Gr1dim and Gr1high subsets of CD11b+Gr1+ splenocytes were isolated from CY+TLRa-treated TB mice on c2d3 of treatment, it was observed that granulocytic CD11b+Gr1hi splenocytes (Figure 5D) had no inhibitory effect upon 4T1-f growth *in vitro*, even with the addition of CpG alone or CpG+IFNγ to culture (Figure 5A-5C column 4). In contrast, the monocytic CD11b+Gr1dim splenocyte fraction (Figure 5D) displayed a reproducible and remarkable potential for tumor cell eradication if reexposed *in vitro* to either CpG alone (Figure 5, image 6 *vs* 10 ***p < 0.0001 and Supplemental Figure S5, image 6 *vs* 10 ***p < 0.0001) or to CpG+IFNγ (Figure 5, image 11 *vs* 15 ***p < 0.0001 and Supplemental Figure S5, image 16 *vs* 20 ***p < 0.0001). These effects were tumoricidal, evidenced by the progressive loss of viable 4T1-f after 48h (Figure 5, image 30) until no viable 4T1-f was detectable at day 7 (Figure 5, image 45). Furthermore, in contrast to purified T-cells, the purified CD11b+Gr1dim fraction appeared to have been at least partially activated prior to culture, since *in vitro* re-exposure merely to CpG was sufficient to trigger anti-tumor activity at least transiently (Figure 5, image 8 *vs* 10 ***p < 0.0001).

In contrast to CY+TLRa-treated mice, assays of splenocytes from untreated TB mice revealed that neither UF splenocytes nor Gr1+ myeloid subpopulations (Gr1hi or Gr1dim) displayed tumoricidal properties, even following *in vitro* exposure to CpG, IFNγ or CpG+IFNγ (Supplemental Figure S5, columns 2 and 3). Therefore, even though CD11b+Gr1dim splenocytes from both untreated and CY+TLRa-treated TB mice displayed MDSC function (Supplemental Figure S4), only treatment with CY+TLRa also licensed the potential for tumoricidal properties in this fraction.

Since our previous analyses (Figure 4 above) demonstrated that the vast majority of CD45^pos^ tumor-infiltrating leukocytes in regressing tumors of CY+TLRa-treated mice were CD11b+Gr1dim and CD11b+Gr1high myeloid cells (Figure 4F-4G), we also sought to evaluate their tumoricidal properties. However, likely due to their highly activated state within regressing tumors, efforts to isolate Gr1dim and Gr1high fractions in a largely viable state were unsuccessful (data not shown). Similar limitations were observed when total regressing tumor digests from CY+CpG-treated mice were subjected to CD45 leukocyte enrichment. In contrast, CD45^pos^ intratumoral leukocytes could be purified in a highly viable state from untreated TB mice. Furthermore, when isolated from the tumor environment, such leukocytes attacked tumor cells *in vitro* for at least 48h in culture, even without *in vitro* exposure to CpG and/or IFNγ (Supplemental Figure S6, image 1 *vs* 2 ***p < 0.0001), suggesting some degree of prior *in vivo* activation even in the absence of treatment. However, this killing was not consistently sustained beyond 48h (data not shown).

### Tumoricidal activation resulting from CY+TLRa treatment requires phagocytic cells and nitric oxide production, and is unaffected by tumor expression of Programmed Death-Ligand 1 (PD-L1)

**Figure 6:**
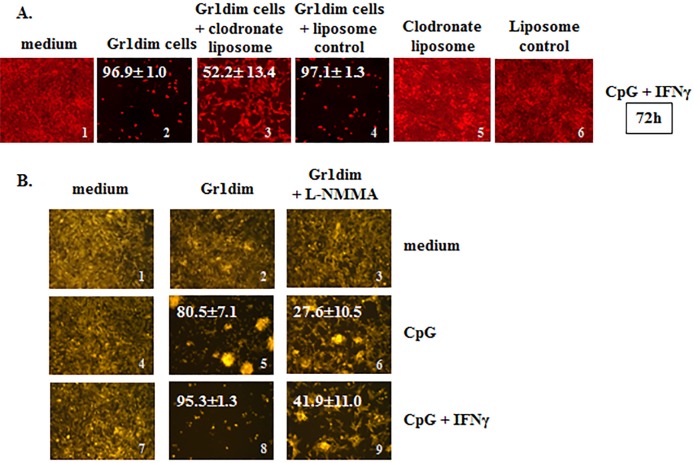
Isolated tumoricidal Gr1dim cells from CY+CpG-treated mice are NO-producing phagocytes **A.** CpG+IFNγ activated myeloid Gr1dim cells from spleens (1×10^5^) of CY+CpG-treated mice were co-cultured with non-irradiated 4T1-f cells (2×10^4^) in the presence or absence of clodronate liposome (25 ng/microwell) or liposomes control at time 0, and tumoricidal activity (% ± SD) was evaluated from 48h to 7 days. Representative photos of the assays at 72h are shown. Data are representative of three technical replicates with similar results. **B.** Representative photos showing the effect of L-NMMA (iNOS inhibitor, 5mM) upon CpG or CpG+IFNγ activated Gr1dim cells isolated from spleens of CY+CpG-treated mice after 48h in culture. Data are representative of three independent experiments. Images without percentages represent confluent monolayers (killing below detection levels). Images were numerically labeled on the bottom right for the purpose of identification. Photos were taken using an EVOS FL Auto imaging system microscope (A) or a Zeiss Axio Observer A1 microscope (B). Statistical analysis presented in results was performed using Student's *t*-Test.

We employed clodronate liposomes to determine if phagocytic cells were required for the observed *in vitro* tumoricidal impacts of CpG+IFNγ-activated Gr1dim cells from the spleens of CY+TLRa-treated mice. Such assays confirmed that the tumoricidal capacity was critically dependent on phagocytic cells, since anti-tumor impacts were significantly reversed in the presence of clodronate but not in the liposome control (Figure 6A, image 2 *vs* 3 ***p < 0.0001, and image 2 *vs* 4, ns *p* = 0.4724). Additional studies based on *in vivo* administration of clodronate liposomes proved to be relatively uninformative due to highly non uniform depletion of intratumoral phagocytic cells (data not shown).

Further mechanistic studies revealed that Gr1dim cells from CY+TLRa-treated mice shared properties classically associated with tumoricidal macrophages, such as the ability to distinguish between malignant and non-transformed cells, killing only the former without MHC or antigen restrictions [17]. CD11b+Gr1dim splenocytes from CY+CpG-treated 4T1 TB mice required only *in vitro* reexposure to CpG to inhibit both 4T1 and CT26 tumor cells, while sparing non-transformed mouse 3T3 fibroblasts (Figure 5 and data not shown). Furthermore, because activation of macrophage antitumor activity can be associated with inducible nitric oxide synthase (iNOS) [61], we also investigated whether Gr1dim myeloid cells from CY+CpG-treated mice employed nitric oxide (NO) to control tumor growth. As previously observed, antitumor activity was evident within 48h following *in vitro* treatment with CpG, and even more so with the combination of CpG+IFNγ (Figure 6B, images 5 and 8). In both cases, this antitumor activity was strongly reduced by the iNOS inhibitor L-NMMA (Figure 6B, images 5 *vs* 6 and 8 *vs* 9 ***p < 0.0001 in both cases), confirming that NO production is at least one mechanism by which Gr1dim myeloid cells from CY+CpG-treated mice can kill malignant cells.

We assessed the impact of IFNγ and/or CpG upon *in vitro* expression of PD-L1 (B7-H1) by 4T1-f cells, since such expression could impair or limit the immune response through checkpoint inhibition. As also reported by others, IFNγ resulted in a strong up-regulation of PD-L1 expression on tumor cells [62] (Supplemental Figure S7). Such a strong up-regulation of PD-L1 did not prevent 4T1 eradication by Gr1dim cells in the presence of CpG+IFNγ (Figures 5 and 6 and Supplemental Figure S5).

### CY+CpG treatment also induces CD11b+Gr1dim cells with tumoricidal potential in naïve, non-tumor-bearing mice

**Figure 7:**
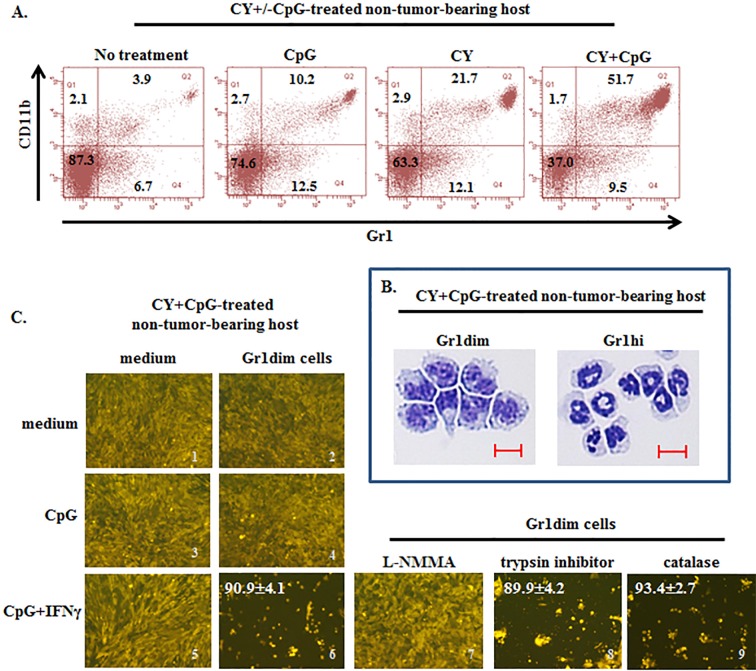
Induction of tumoricidal CD11b+Gr1dim cells in non-tumor-bearing mice by CY+CpG treatment **A.** Induction of double-positive CD11b+Gr1+ cells in spleens of non-TB mice treated with CpG, CY or CY+CpG compared to untreated naïve mice analyzed by flow cytometry. Numbers in the plots correspond to percentages of cells per quadrant. **B.** Representative pictures of cytospin analyses of isolated Gr1dim and Gr1hi cells from spleens of CY+CpG-treated non-TB mice. Scale bar, 10 μm. **C.** Evaluation of tumoricidal properties in Gr1dim cells isolated from CY+CpG-treated non-TB hosts. Representative photos of 4T1-f growth in the presence or absence of splenic Gr1dim cells from CY+CpG-treated non-TB mice stimulated *in vitro* with CpG or CpG+IFNγ. In additional wells containing Gr1dim splenocytes, 4T1-f and CpG+IFNγ, L-NMMA (iNOS inhibitor, 5mM), trypsin inhibitor (serine protease inhibitor, 4,000 U/ml) or catalase (ROS inhibitor, 4,000 U/ml) was added individually. Tumoricidal effect of Gr1dim cells was evaluated as in Figure 5A-5C. The percentage of tumor growth inhibition is denoted in white numbers (top left). Images without percentages represent confluent monolayers (killing below detection levels). Images were numerically labeled on the bottom right for the purpose of identification. Data are representative of 3 independent experiments. Three to five mice were pooled per experiment. All the studies were performed with cells isolated after mice received one round of treatment (c1d7). Representative photos shown in C correspond to 48h in culture. Photos were taken using a Zeiss Axio Observer A1 microscope. Statistical analysis for 4T1-f killing assay presented in results was performed using Student's *t*-Test.

CD11b+Gr1+ cells in naïve mice, designated immature myeloid cells (IMC), mainly reside in bone marrow and spleen, and in the absence of tumor are not induced to differentiate into MDSCs (Supplemental Figure S1D and Figure 7A) [49, 63]. We sought to determine whether CY+CpG treatment required tumor-induced monocytic MDSCs as a source of tumoricidal monocytes, or whether CY+CpG could also generate Gr1dim tumoricidal monocytes in non-TB mice. Naïve mice were treated with a single cycle of CY, CpG or CY+CpG and were sacrificed at the end of this cycle (c1d7). Gr1dim and Gr1hi myeloid subpopulations appeared intrasplenically following CY+CpG treatment which were remarkably enriched both in frequency and absolute numbers (Figure 7A-7B and data not shown). CY or CpG alone was considerably less effective. We confirmed that *in vivo* CY+CpG-induced Gr1dim cells could be activated *in vitro* by CpG+IFNγ to significantly control 4T1-f growth at 48h (Figure 7C, image 5 *vs* 6 ***p < 0.0001). This anti-tumor impact was primarily iNOS-mediated as indicated by the inhibitory effect that L-NMMA had on Gr1dim cells, blocking their CpG+IFNγ-induced tumoricidal properties (Figure 7C, 6 *vs* 7 ***p < 0.0001). The antitumor effect of Gr1dim cells did not involve serine proteases or reactive oxygen species (ROS) since the addition of blockers of these mechanisms (trypsin inhibitor or catalase, respectively) did not revert the killer properties of Gr1dim cells (Figure 7C, image 6 *vs* 8 and 9; ns *p* = 0.6852 and ns *p* = 0.2407, respectively) even at high concentrations (data not shown). The finding that CY+CpG treatment induces tumoricidal myeloid cell precursors even in non-TB mice demonstrated that preexisting tumor-induced MDSCs are not an essential source of tumoricidal myeloid precursors during CY+CpG treatment. Furthermore, CY+CpG's immunomodulatory impacts on host cells occurred even in the complete absence of the tumor-bearing state.

### Human monocytes can be activated by combined TLRa and IFNγ to acquire tumoricidal properties

To evaluate whether the tumoricidal profile achievable by mouse myeloid cells could also be achieved in the human setting, we performed a series of *in vitro* assays in which human peripheral blood monocytes from four healthy donors were co-cultured with the fluorescent (f) cell lines MDA-MB-231-f (human breast cancer), 4T1-f (murine breast cancer), or 3T3-f (non-transformed mouse fibroblasts) in the presence or absence of rhIFNγ, TLR4 agonist LPS, and TLR8 agonist resiquimod (R848). These agents were selected because of their strong capacity to activate human monocytes and myeloid dendritic cells, as well as because of the lack of TLR9 and TLR7 expression on human monocytes [64]. In addition, rhGM-CSF was added to the cultures to promote monocyte viability. Assays were evaluated from 24 hours to 7 days.

**Figure 8:**
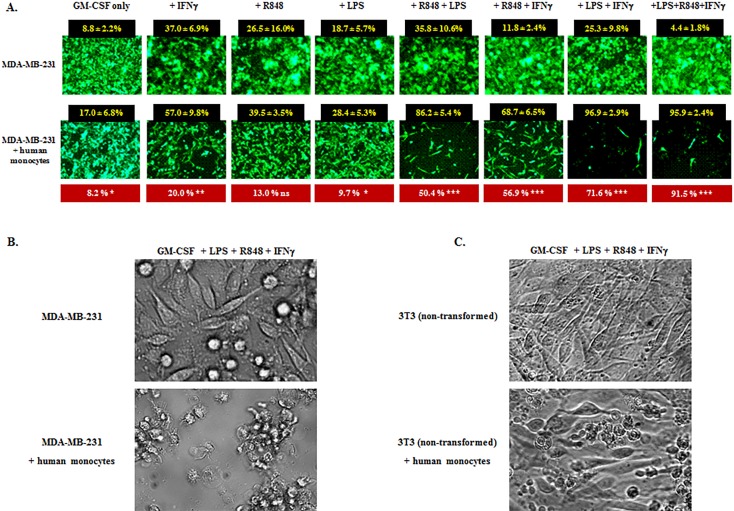
IFNγ and TLRa promote tumor-inhibitory properties in human monocytes **A.** Human breast cancer cell line MDA-MB-231 tGFP (2×10^4^) was cultured in the presence or absence of enriched human monocytes from healthy donors (1×10^5^), and IFNγ, resiquimod (R848) and/or LPS were variably added to the assays at time 0; all wells received GM-CSF to promote sustained viability. Upper panel displays treatment with the above factors but without monocytes (M-); lower panel displays the same factors but also with human monocytes (M+). The 96h time point is shown, representative of four healthy monocyte donors. For each factor or factor combination, % tumor inhibition was enumerated for six non-overlapping HPF with or without monocytes (black boxes). Monocyte-attributable inhibition of MDA-MB-231 cells (shown in red boxes) was calculated for each factor set by subtracting background inhibition in the absence of monocytes. The red boxes also indicate the degree of significance between M- and M+ groups for each factor analyzed by unpaired Student's *t*-Test. Confluence of MDA-MB-231 cells was variably delayed by the tested factors even in the absence of monocytes but to a significantly greater degree when monocytes were also present (* *p* < 0.05, **p < 0.01, ***p < 0.001), the exception being wells which received only GMCSF+R848 (ns, non-significant). **B.** Bright field examination of MDA-MB-231 cells exposed to GMCSF+IFNγ+LPS+R848 either without (upper panel) or with (lower panel) co-exposure to human monocytes (same treatments as far right group in A). Upper panel shows nearly confluent tumor outgrowth whereas lower panel shows largely eradicated MBA-MB-231 cells with prominent acellular spaces and monocytes clustering on residual tumor cells. **C.** Same as B except that the target is 3T3 non-transformed mouse fibroblasts. 3T3 cells achieve near confluence regardless of GMCSF+IFNγ+LPS+R848 exposure without monocytes (upper panel) or with monocytes (lower panel), the monocytes failing to form clusters with the 3T3 cells.

Non-irradiated MDA-MB-231-f cells seeded at 20,000 cells per microwell in medium supplemented only with GM-CSF reached near confluence by 96 hours in the absence of monocytes (Figure 8A upper panel). When human monocytes (100,000 cells per microwell) were also added in the absence of both IFNγ and TLRa, modest but significant tumor inhibition was observed (Figure 8A lower panel leftmost group). In contrast, while the addition of IFNγ and/or TLRa without monocytes displayed varying background inhibition of MDA-MB-231-f cells (Figure 8A upper panel), the additional inclusion of monocytes could markedly and significantly boost tumor inhibition, especially when combinations of IFNγ and/or TLRa were included (Figure 8A lower panel). Examination of groups under bright field confirmed that areas lacking fluorescent MDA-MB-231-f cells were denuded of tumor, with monocytes clustering upon the few remaining tumor cells (Figure 8B). Similarly, human monocytes activated in the presence of IFNγ and TLRa also strongly inhibited 4T1-f growth (data not shown), indicating that the antitumor effect was not MHC nor antigen restricted. In contrast, such activated monocytes spared non-transformed 3T3 fibroblasts even in the presence of GM-CSF, IFNγ, LPS and R848 (Figure 8C), paralleling our observations for CY+TLR-induced tumoricidal mouse myeloid cells. Similar findings were observed for all four monocyte donors, suggesting that the CY+TLRa therapeutic strategy has the potential to be operational in humans.

## DISCUSSION

We have demonstrated in these investigations that optimally designed chemoimmunotherapy can be used to eradicate aggressive syngeneic mouse tumors after unresected primary tumors have already reached 50-100 mm^2^ dimension, innumerable metastases have already been established, and life expectancy is a matter of days. The effectiveness of repetitive CY+TLRa treatment for a wide range of tumors of multiple tissue origins, different strains, widely divergent MDSC content and the virtual absence of treatment intolerance make this strategy a promising candidate for cancer therapy. Our observations in human monocytes confirmed that a parallel tumoricidal mechanism can also be achieved in humans.

The reason for CY's highly significant therapeutic superiority to other tested chemotherapeutic agents in conjunction with TLRa is not readily apparent, particularly since two additional alkylating agents, temozolomide and oxaliplatin, proved ineffective (data not shown). CY+TLRa treatment generated CD11b+Gr1dim tumoricidal precursors even in naïve non-TB mice (Figure 7), confirming that this is a direct impact on normal host cells rather than an indirect effect resulting from tumor cytoreduction. Interestingly, two tested agents, 5-FU and the receptor tyrosine kinase inhibitor sunitinib, were well tolerated in conjunction with TLRa and furthermore depleted splenic MDSCs in the 4T1 and CT26 models even more than CY (data not shown), yet in contrast to CY+TLRa did not reverse tumor progression (Supplemental Figure S2C and data not shown). Finally, since combining gemcitabine (GEM) with TLRa in TB mice was unexpectedly poorly tolerated compared to GEM alone (Supplemental Figure S2B and S2C), we performed extensive ancillary experiments in which GEM and/or TLRa were dose reduced in search of a better tolerated regimen; nonetheless, efficacy comparable to CY+TLRa was not observed at any tested dose of GEM+TLRa (data not shown).

Remarkably, agents that were themselves ineffective in combination with TLRa, including 5FU and docetaxel, could be coadministered with CY+TLRa to achieve well-tolerated tumor regressions equivalent to CY+TLRa alone (data not shown). Therefore, even though these additional agents did not synergize from the immunotherapy perspective, they could still be co-administered without detriment to CY+TLRa's immunopotentiating impact.

It is also not readily apparent why definitive treatment with CY+TLRa for some tumor models required paired rather than single TLRa. Because such dual TLRa stimulation of MyD88 and TRIF pathways markedly increases host production of IL-12 [55], we examined whether CY+TLRa treatment would be therapeutically abrogated in IL-12 knockout mice, particularly in models requiring two TLRa. Surprisingly, whether requiring one or two TLRa for cure, tumor models were equally curable by CY+TLRa in WT as well as in both IL12p35 knockout and IL12p40-knockout syngeneic mice (data not shown).

One of the major findings of our studies is that it is routinely possible to eradicate even advanced, highly metastatic tumors solely by modulating the endogenous immune response through the administration of CY+TLRa, with no need to provide tumor-antigen vaccinations or adoptive transfer of T-cells. Intratumoral administration of TLRa proved completely unnecessary, and even remote s.c. administration was highly effective, rendering this treatment potentially applicable to the majority of patients who do not have easily accessible tumors for repeated intralesional injections. Eradication of tumors depended upon a natural CD4+ and CD8+ T-cell presence in all our screening models, as administration of depleting anti-CD4/CD8 mAbs prevented CY+TLRa-mediated durable tumor rejection (Figure 2A). While weekly CY+TLRa treatment predictably resulted in partial depletion of tumor-reactive IFNγ-secreting T-cells (Figure 3), such treatment also produced an even greater body wide progressive depletion of Tregs, especially intratumorally, where as early as c2d3 Foxp3+ T-cells were virtually undetectable (Figure 4).

Even though there was evidence for immunological memory, the endogenous anti-tumor T-cell response present in cured mice proved to be relatively ineffective against tumor rechallenge in the absence of CY+TLRa retreatment, itself a T-cell dependent process. It is likely that repetitive chemotherapy limited the sustained numeric expansion of endogenous effector T-cells, rendering the T-cells co-dependent upon other host cells for definitive tumor rejection. Importantly, even CY-depleted anti-tumor T-cells proved sufficient in conjunction with other host elements not only to mediate complete tumor rejection, but also to prevent subsequent spontaneous tumor relapse.

In aggregate, our results strongly point to activated CD11b+Gr1dim myeloid host cells as the final arbiters of CY+TLRa-mediated tumor rejection, with small numbers of endogenous T-cells playing at least a critical helper role. Although IFNγ-producing, CD4+ and CD8+ host T-cells were crucial for the effectiveness of the treatment (Figure 2A), and although tumor-specific IFNγ-producing T-cells were present in treated mice (Figure 3A-3B), isolated T-cells did not display demonstrably sustained tumoricidal nor tumoristatic effects even after *in vitro* exposure to exogenous IFNγ and TLRa (Figure 5). Furthermore, even though durable tumor eradication by CY+CpG treatment was T-cell dependent (Figure 2), T-cell absolute numbers were reduced both peripherally and intratumorally compared to those observed in untreated TB mice (Figure 4). In contrast, CD11b+Gr1+ host myeloid cells remained the dominant host constituent in the regressing tumors of CY+CpG-treated 4T1-bearing mice (Figure 4) and, as importantly, CD11b+Gr1dim cells from CY+TLRa treated TB or even naïve mice became highly tumoricidal upon *in vitro* reexposure to exogenous CpG, with the tumoricidal state extending to 7 days if reexposure to IFNγ was also included (Figures 5, 6, 7). Such tumor cell destruction was significantly blocked by clodronate-induced elimination of phagocytic cells as well as by NO inhibition (Figure 6).

The potential of tumoricidal myeloid cells to serve as final mediators of tumor rejection has recently become more appreciated; for example, Beatty et al. demonstrated for both mouse and human pancreatic carcinoma a likely pivotal role of tumor-infiltrating myeloid cells activated to a tumoricidal state through an agonal CD40 mAb [14, 65]. While the mechanism by which tumoricidal myeloid cells recognize tumor cells remains incompletely understood, it bypasses recognition of specific antigens and it is not MHC restricted [60]. In classical studies by Fidler's group, co-passage of tumor cells with cytolytic T-cells (CTL) led to complete tumor resistance to T-cell mediated cytolysis within several passages, whereas multiple co-passages of tumor cells with tumoricidal myeloid cells did not lead to tumor resistance [16]. Therefore, while tumor down-regulation of antigen/MHC complexes represents a formidable escape mechanism for T-cell mediated target lysis, it does not render tumor cells resistant to tumoricidal myeloid effector cells. In addition, PD-L1 expression by tumor cells, a check-point for T-cell-mediated tumor rejection, did not impede myeloid cell-mediated tumor killing in our *in vitro* assays.

Much evidence supports that tumoricidal myeloid cell recognition of tumors is mediated through surface lectins which bind to aberrantly glycosylated proteins on the transformed cell's surface [60]. Such aberrant glycosylation is pathognomonic for malignant cells, consistent with activated myeloid cells’ ability to distinguish malignant cells from non-transformed cells and to kill only the former [66–70]. In the present study, CY+TLRa treatment *in vivo* followed by IFNγ and TLRa reexposure *in vitro* generated what appears to be a maximum tumoricidal state in Gr1dim (monocytic) myeloid cells, effectively obliterating actively dividing, non-irradiated tumor cells for at least seven days in culture.

At the time points examined *in vitro,* both MDSC function and tumoricidal function were concentrated in the Gr1dim monocytic subset of CD11b+Gr1+ myeloid cells (Supplemental Figures S4-S5 and Figures 5–6). This raises the possibility that CY+TLRa treatment modulates a transition from MDSC function to tumoricidal function in naturally MDSC-rich tumor models such as 4T1 and CT26. However, MDSC-poor models such as Panc02 also responded to CY+TLRa therapy. This is likely explained by our observations that treatment of even non-TB mice with a single cycle of CY+CpG induced *de novo* expansion of CD11b+Gr1dim splenocytes with tumoricidal potential. Similarly, in mice bearing Panc02 tumors, CY+CpG+pIC treatment induced a *de novo* surge of both CD11b+Gr1high and CD11b+Gr1dim cells intratumorally as well as intrasplenically (data not shown). Such findings support the hypothesis that the repetitive bone marrow rebounds triggered by weekly CY include bodywide extramedullary distributions of CD11b+Gr1+ cells, effectively delivering fresh Gr1dim tumoricidal myeloid precursors into tumor deposits. Once situated intratumorally, the tumoricidal myeloid precursors can be activated by exogenous TLRa in tandem with IFNγ supplied by the endogenous anti-tumor T-cell response. The observation that CY+TLRa fails to be curative if weekly CY is replaced by daily CY (Supplemental Figure S3B), likely reflects a therapeutic requirement for bone marrow extramedullary replenishments which are lacking in the steady state leukopenia resulting from daily chemotherapy.

It is not yet clear why several tumor models are stabilized by treatment with CY+TLRa so long as the treatment persists, yet fail to achieve durable regressions. This pattern of partial disease resistance is especially interesting in the case of B16 melanoma because B16 is highly sensitive to tumoricidal macrophages *in vitro*, and remains so during serial co-culture with tumoricidal macrophages [16]. This is in contrast to serial co-culture with CTL, which progressively results in B16 resistance to CTL [16]. The observed pattern of therapeutic stabilization that ends with cessation of CY+TLRa treatment closely resembles the treatment resistance observed when 4T1 or other responsive tumors are treated with CY+TLRa either in nude mice or in T-cell depleted WT mice (Figure 2 and not shown). We are testing the hypothesis that bolstering the endogenous T-cell response will improve the therapeutic outcome in partially refractory tumor models.

The ability of mouse myeloid cells to effectively mediate tumor cell rejection raises the question of what treatment can best elicit the equivalent properties in human myeloid cells. Major differences in TLRa usage between mice and humans are evident. For example, TLR8 is dysfunctional in mice [71] and TLR9 and TLR7 are not expressed by human myeloid cells [64]. The strong *in vitro* tumoricidal activation of mouse myeloid cells by treatment with CpG+IFNγ can, however, be replicated in human monocytes by exposure to combinations of TLR8 agonist (resiquimod), TLR4 agonist (LPS) and/or IFNγ (Figure 8). Furthermore, human monocytes activated to a tumoricidal state continued to spare non-transformed cells (Figure 8C). We are conducting a clinical trial to determine if patients with advanced cancers tolerate and respond to combinational treatment with CY and the novel TLR8 agonist motolimod as meaningfully as mice treated with CY+CpG ODN 1826.

In summary, we have shown for multiple tumor models that the endogenous T-cell response to tumor may not require vaccinations or *ex vivo* expansion to mediate tumor rejection. Within the course of CY+TLRa treatment, T-cells provide an essential helper function which likely orchestrates, in conjunction with exogenous TLRa, activation of tumoricidal myeloid cells. Given the apparent inability of tumor cells to escape from tumoricidal myeloid recognition, it appears to be a therapeutic advantage for the host that myeloid cells rather than T-cells serve as the final mediators of tumor killing. Similar ready inducibility of tumoricidal function in human peripheral monocytes renders the CY+TLRa treatment algorithm a promising strategy to be applied to the therapy of human malignancies.

## MATERIALS AND METHODS

### Ethics statement

Investigation has been conducted in accordance with the ethical standards and according to the Declaration of Helsinki and according to national and international guidelines and has been approved by the authors’ institutional review board. Research involving human participants has been approved by the Mayo Clinic Institutional Review Board, IRB 09-000263. Informed consent has been obtained.

### Mice

All experiments were performed with 8- to 10-week old female BALB/c wild type (WT) mice purchased from NCI Frederick (Frederick, MD). C57BL/6 wild type mice and IFNγ knockout (KO) mice on the BALB/c background (C.129S7(B6)-Ifngtm1Ts/J) were purchased from Jackson Laboratory (Sacramento, CA). Athymic nude mice (BALB/c nu/nu mice (C.Cg/AnNTac-Foxn1nu NE9) were purchased from Taconic (Oxnard, CA). All mice were housed in the Natalie Schafer animal facility at the Mayo Clinic in Arizona and kept under specific pathogen-free conditions. All protocols were approved by the Mayo Clinic Institutional Animal Care and Use Committee (IACUC).

### Antibodies and reagents

Chemotherapy agents were obtained from the Mayo Clinic in Arizona chemotherapy pharmacy or from Sigma-Aldrich. CpG oligodeoxynucleotides 1826 (CpG) were purchased from Oligos ETC (Wilsonville, OR). Polyinosinic:polycytidylic acid (pIC) was obtained from Sigma. Recombinant mouse IFNγ was purchased from Peprotech (Rocky Hill, NJ). The depleting mAbs anti-CD4 (GK1.5) and anti-CD8a (2.43), and the respective isotype control (purified Rat IgG control) were obtained from Leinco Technologies INC (St. Louis, MO).

### Cell lines

The 4T1 mammary tumor cell line was a gift from Suzanne Ostrand-Rosenberg (University of Maryland). The Panc02 pancreatic adenocarcinoma cell line was kindly provided by Dr. M.A. Hollingsworth. CT26 colon carcinoma was purchased from ATCC. The 4T1-luc2-tdTomato cell line was purchased from Caliper. Cell lines were verified at the end of the experiments (6/1/2015) as being entirely of mouse origin from the BALB/c or C57BL/6 strains and no mammalian interspecies contamination was detected using a panel of microsatellite markers for genotyping (IDEXX BioResearch, Columbia, MO). All cells were determined to be free of mycoplasma (IDEXX). BM185 cell line was derived from bone marrow from an acute lymphoblastic leukemia model, originally provided by D. Kohn (University of Southern California, Los Angeles, CA). MDA-MB-231 cell line was originally obtained from Imperial Cancer Research Fund cell culture core (London, UK). Cells were stably transfected for tGFP by Innoprot (Derio-Bizkaia, Spain). IDEXX verification confirmed that the cell line was of human origin and no mammalian interspecies contamination was detected. The cell line was consistent with the ATCC MDA-MB-231 cell line. Cell lines were tested and confirmed negative for mycoplasma and viral pathogens. All 4T1 cell lines, CT26, Panc02, BM185 and MDA-MB-231 tGFP cell lines were cultured in RPMI 1640 (Lonza Walkersville, MD) supplemented with heat inactivated FBS (Gibco Carlsbad, CA) at final concentration 10%, 2 mM L-glutamine (Lonza), 50 uM 2-ME (Sigma-Aldrich INC St. Louis, MO), 100 U/ml penicillin and 100 μg/ml streptomycin (Lonza) (cRPMI). CT26 tGFP cells were grown in cRPMI 1640 supplemented with G-418 sulfate 500 ug/ml (Adipogen San Diego, CA), tGFP-3T3 Green and 3T3 NIH Red cell lines were grown in DMEM (Gibco) supplemented with heat inactivated FBS at final concentration 10%, 2 mM L-glutamine (Lonza), 50 uM 2-ME (Sigma-Aldrich INC St. Louis, MO), 100 U/ml penicillin and 100 μg/ml streptomycin (Lonza) and 3ug/ml Puromycin (InvivoGen San Diego, CA). All cells were cultured and maintained at 37°C in 5% CO2. Cells were used for *in vivo* injection and/or *in vitro* assays once they were in log phase of growth and reached about 75% confluence.

### Tumor mouse models

For tumor growth experiments and evaluation of the effect of chemotherapy and TLRa treatments, cells were injected as follows: For ectopic models, 4T1 cells were injected subcutaneously (s.c) into the right flank of syngeneic BALB/c or nude mice at a concentration of 1×10^6^ cells/0.1 ml PBS. Panc02 cells were injected s.c. into the right flank of syngeneic C57BL/6 mice at a concentration of 5×10^6^ cells/0.1 ml PBS cells. CT26 cells were injected s.c. into the right flank of syngeneic BALB/c mice at a concentration of 2×10^6^ cells/0.1 ml PBS. For 4T1 orthotopic model, 5×10^5^ cells/0.1 ml PBS were injected into the left fourth inguinal mammary fat pad of BALB/c mice. For 4T1 tumor rechallenge experiments, long-term tumor-free BALB/c mice (for one to three months) were ectopically injected s.c. into the left flank with 4T1 cells (0.5-1.0×10^6^/0.1 ml PBS). Aged-matched naïve mice were also injected with same number of 4T1 cells as a control group. Tumors were measured with a caliper and tumor size was determined as the bidimensional product of the longest perpendicular length and width measurements (mm^2^).

### Screening of chemotherapeutic agents

In preliminary experiments groups of non-TB mice received graded doses of each tested agent weekly for three cycles (comparable to 21-28 day cycles in humans) to determine the maximum tolerated dose (MTD) of each agent given on this basis. The chemotherapy agents were then tested at their established MTD in TB mice with or without CpG and pIC.

### Standardized cyclophosphamide (CY) + TLRa schedule subsequent to preliminary studies

For 4T1 models, tumors were allowed to grow until primary tumor reached a size between 50-100 mm^2^ (14-17 days after tumor challenge). For other models, initiation of treatment varied according to the growth kinetics of the model; PanC02, 26-35 days and CT26, 8 days. For all mouse tumor models CY+TLRa treatment was administered in weekly cycles for 7 cycles. Mice were injected i.p. with CY (200g/kg) on day 0, and with CpG alone (4T1 and CT26) or CpG plus pIC (Panc02) (each at 5 mg/kg) on day 3 of each cycle coincident to the leukocyte nadir, followed by a 4-day rest period to allow bone marrow recovery before the next CY dose was given. Tumor size was recorded on d0 before the first CY injection, and then monitored at least weekly. Mice were sacrificed at different time points to evaluate the effect of treatments on TB mice; otherwise, mice were euthanized when primary tumor reached the threshold size of 250 mm^2^ or earlier if mice appeared moribund, in accordance with the Institutional Animal Care and Use Committee (IACUC) guidelines. In naïve BALB/c mice (non-TB), CY, CpG or CY+CpG was administered for a single cycle (one week), following the same CY+CpG scheme used for 4T1 tumor model. Mice were studied at the end of the first cycle of treatment (c1d7).

### Lung metastasis analysis

4T1 TB mice were sacrificed at different time points after 4T1 challenge. Lungs were infused with India ink (15% in PBS) *via* the trachea, resected and then washed by placing them into 3 ml of Fekete's solution (100 mL of 70% alcohol, 10 mL of 10% buffered formalin, and 5 ml of glacial acetic acid) for 5 minutes. Lung metastases appeared as white spots as they did not retain the India ink solution after bleaching in Fekete's solution while normal tissue remained black. Lung metastases were enumerated and pictures were taken using a Stereo Discovery V8 stereomicroscope.

### CD4+ and CD8+ cell depletion

CD4+ or CD8+ cells were depleted in WT BALB/c or C57BL/6 mice by injecting 125ug in a volume of 200 ul PBS of anti-CD4, anti-CD8, or IgG (isotype control) intraperitoneally (i.p.). The depleting mAbs and isotype control were initially given 4 days before 4T1 inoculation, and then administered every 8 days throughout the 7 cycles of the CY+TLRa treatment. Effectiveness of the T-cell depletion procedure was confirmed by cytometric analyses of CD4+CD3+ and CD8+CD3+ populations in spleens of non-experimental mice.

### *In vivo* treatment with rmIFNγ

8- to 10-week old female nude mice were injected i.p. with 10 μg of rmIFNγ (Peprotech, Cat No. 315-05). Injections were performed the same day of CpG injections, during the CY plus CpG treatment scheme.

### Spleen, tumor and LN processing

Spleens were excised and directly mashed on 40 μm nylon cell strainers to get single cell suspensions followed by red blood cell lysis. Tumors were resected, minced and underwent enzymatic digestion (collagenase type IV, DNase and hyaluronidase, Sigma) for 2h at room temperature to release tumor-infiltrating leukocytes. Tumor digest was passed through a 40 μm nylon cell strainer to remove persistent tumor debris and washed with PBS. For LN processing, in the case of TB mice, axillary, brachial and inguinal tumor-draining lymph nodes were excised, pooled and directly smashed on 40 μm cell strainer to obtain single cell suspensions followed by red blood cell lysis. The same procedure was applied for LN obtained from naïve mice in which axillary, brachial and inguinal LN from both left and right sides were pooled for analyses. Total cell count was performed for spleen, tumor, and total lymph node cell suspensions samples and viability was evaluated by trypan blue.

### Total T-cell adoptive transfer

Total T-cells (CD3+ cells) were enriched by negative selection (Dynabeads untouched mouse T-cells, Invitrogen/Molecular Probes, Eugene OR) from total splenocytes obtained from naïve BALB/c and IFNγ KO mice following manufacture's instruction. In brief, total splenocytes after red cell lysis were adjusted to a concentration of 1×10^6^ cells in 1 ml isolation buffer, cells were incubated with antibody mix (against B-cells, monocytes/macrophages, NK cells, dendritic cells, erythrocytes, and granulocytes) for 20 min at 4°C. Cells were washed with isolation buffer, and then Depletion Dynabeads were added to the cells. After 15 min incubation at room temperature, cells were washed and magnetic separation was performed. Total T-cells were present in the supernatant. Enriched T-cell preparations were analyzed by flow cytometry using anti-CD3, anti-CD4 and anti-CD8 mAbs and 99% cell purity was confirmed prior to adoptive transfer. Total T-cells were injected into the tail vein of BALB/c nude mice in a concentration of 30×10^6^ in 0.1 ml PBS eight days before 4T1 challenge. Successful T-cell adoptive transfer was confirmed in randomly selected mice by flow cytometric analysis of CD4+CD3+ and CD8+CD3+ populations in spleen and lymph node samples.

### CD8+ and CD4+ cell enrichment

CD8+ and CD4+ cells were isolated by negative selection (Dynabeads untouched mouse CD8+ cells or CD4+ cell kit, respectively; Invitrogen) from total splenocytes or total lymph node single cell suspensions, following manufacture's instruction. In brief, total splenocytes or LN cells after red cell lysis were adjusted to a concentration of 5×10^7^ cells in 0.5 ml isolation buffer and 0.1 ml FBS was added. Cells were incubated with 0.1 ml antibody mix for CD8+ or CD4+ cells for 20 min at 4°C. Cells were washed with isolation buffer, and then 1 ml Mouse Depletion Dynabeads was added to the cells. After 15 min incubation at room temperature, cells were washed and magnetic separation was performed. Total CD8+ or CD4+ cells were present in the supernatant. Enriched T-cell preparations were analyzed by flow cytometry using anti-CD3, anti-CD4 and anti-CD8 mAbs and > 90% cell purity for either CD8+ or CD4+ T-cell subpopulation was confirmed.

### IFNγ ELISpot assay

IFNγ production in enriched subpopulations of T-cells was evaluated by ELISpot using mouse IFNγ ELISpot^plus^ kit (3321-4APW, Mabtech Cincinnati, OH) following the manufacturer's instructions. In brief, freshly isolated T-cells (CD4+ or CD8+) from spleens or LN of naïve, untreated and CY+CpG-treated 4T1 TB mice were washed twice with cRPMI. Cells were filtered through a 40 μm nylon cell strainer. Enriched CD8+ or CD4+ cells were added in a concentration of 1×10^5^ cells in 100 ul cRPMI media to the ELISpot plates at time 0. As stimuli, irradiated 4T1 or BM185 tumor cell lines (1×10^4^ rads) were added in a concentration of 1×10^5^ cells in 100 ul cRPMI also at time 0. Plates were incubated for 48 h at 37°C degrees and 5% CO2. ELISpot analysis was performed using a Zeiss ELISpot reader (Thornwood, NY) with KS ELISpot software 4.9. CD8+ or CD4+ cells in cRPMI alone were used as a negative control. Each sample was run in triplicates for each biological assay. IFNγ production was analyzed as number of IFNγ spots per 1×10^5^ enriched T-cells.

### Gr1hi and Gr1dim host myeloid cell enrichment

CD11b+Gr1hi and CD11b+Gr1dim cells were enriched by using Myeloid-Derived Suppressor Cell Isolation Kit, mouse (130-094-538, Miltenyi Biotec) following the manufacturer's instructions. In brief, splenic total single cell suspensions were washed with isolation buffer (0.5% BSA and 2mM EDTA in PBS, pH 7.2). Gr1hi (Gr1hiLy6G^pos^) cells were positively selected by incubation with anti-Ly6G-biotin and anti-biotin microbeads, and then magnetic cell separation was performed, using LS columns (Miltenyi Biotec). The effluent (the pre-enriched Gr1dimLy6G^neg^ fraction) was incubated with anti-Gr1-biotin and streptavidin microbeads, and then magnetic separation was performed for positive selection of Gr1dim cells, using LS columns (Miltenyi Biotec). Flow cytometric analysis using anti-CD11b, anti-Gr1 and anti-CD3 mAbs confirmed at least 90% purity for Gr1dim cells (∼10% Gr1hi cells and < 0.5% CD3+ cells) and 75% purity for Gr1hi cells (∼25% Gr1dim cells).

### Cytospin analysis

To perform morphological studies of isolated Gr1dim cells and Gr1hi cells from spleens of CY+CpG-treated mice, cytospin preparations (5×10^4^ cells/100ul 5% FBS) of enriched cells were stained with Diff-Quik (modified Giemsa) (Siemens, cat No. B41321A). Photos were taken at 400x magnification using an EVOS xl microscope.

### CD45 cell enrichment for tumor digests

Tumor-infiltrating leukocytes released by enzymatic digestion from resected 4T1 tumors of TB mice were subjected to CD45 enrichment by positive selection (130-052-301, CD45 MicroBeads mouse kit, Miltenyi Biotec), following manufacture's instruction. In brief, total tumor-infiltrating leukocytes were adjusted to 1×10^7^ cells in 90 ul isolation buffer (0.5% BSA and 2 mM EDTA in PBS, pH 7.2), and 10 ul CD45 MicroBeads were added. Cells were incubated for 15 min at 4°C, washed and subjected to magnetic separation, using LS columns (MACS separation Columns, Miltenyi Biotec Auburn, CA). The CD45-magnetically labeled fraction was flushed out from the columns and collected cells were washed and resuspended in cRPMI. Cell viability was evaluated by trypan blue. Flow cytometric analysis using APC/Cy7-conjugated anti-CD45 mAb confirmed > 90% purity.

### Flow cytometry studies

Single cell suspensions were incubated with Fc Receptor blocking solution (20 ml of FACs buffer, 0.5 mg of purified Rat anti-mouse CD16/CD32, and 5 mg of mouse IgG). Cells were adjusted to a concentration of ≤1×10^6^ cells in 100ul of FACs buffer (PBS, 10% FBS, 0.1% NaN3 sodium azide) and added to 96-well round-bottom plates. For surface staining, cells were treated with the following mouse monoclonal antibodies: APC/Cy7-conjugated anti-CD45 (30-F11, Biolegend), Alexa Fluor 700-conjugated anti-CD11b (M1/70, eBiosciences), fluorescein isothiocyanate-conjugated anti-Gr1 (RB6-8C5, BD), Brilliant Violet 421-conjugated anti-CD3 (17A2, Biolegend), Alexa Fluor 700-conjugated anti-CD4 (RM4-5, BD Pharmingen), fluorescein isothiocyanate-conjugated anti-CD8 (53-6.7, BD Pharmingen), Brilliant violet 421-conjugated anti-PD-L1 (10F.9G2, Biolegend) or the corresponding matched-isotype control. Additionally, for intracellular Foxp3 determination, cells were washed and incubated with fixative/permeabilization buffer (eBioscience), followed by the addition of phycoerythrin-conjugated anti-Foxp3 mAb (FJK16s, eBioscience) or isotype control (EBR2a, eBioscience). For Figure 4, total numbers of myeloid CD11b+Gr1+ subpopulations (CD11b+Gr1dim and CD11b+Gr1hi), and T-cells subsets (T_eff_ cells, CD3+Foxp3^neg^; Tregs, CD3+Foxp3^pos^) present in spleen were determined based on total cell counts and the proportions of the different subpopulations obtained by cytometric analyses. For the study of tumor digests, leukocytes were identified by CD45 positive staining. Gated CD45^pos^ cells were then analyzed for the expression of CD11b, Gr1, CD3 and Foxp3 markers to determine the levels of double positive CD11b+Gr1dim, CD11b+Gr1hi, CD3+Foxp3^neg^ and CD3+Foxp3^pos^ cells. Total cell numbers for each cellular subpopulation were determined based on manual total leukocyte counts and the proportions of each cellular subset according to flow cytometric studies. In addition, absolute numbers of cellular subsets were divided by tumor volume (cells/mm^3^). Cells were analyzed on a LSRFortessa machine (BD Bioscience) using FACsDiva software (BD Biosicience).

### *In vitro* tumor killing assay

To evaluate the tumoricidal properties of splenic cells and tumor-infiltrating leukocytes (CD45+ cells) from CY+CpG-treated (c2d3, 2h post CpG injection) and untreated 4T1 TB mice, we devised a 7d *in vitro* tumor-killing assay mainly employing as a target fluorescent 4T1-tdTomato (4T1-f) cells. To emulate *in vitro* the aggressive *in vivo* 4T1 tumor growth, non-irradiated 4T1-f cells were used when they were in log phase of growth and displayed about 75% confluence. These non-irradiated cells were added directly to the plates. 4T1-f cells were plated in 96-well flat-bottom plates at a concentration of 2×10^4^ cells in 0.1 ml of cRPMI media. The plates were incubated for 20-60 min at 37°C degrees and 5% CO2 to allow 4T1-f cells to adhere to the plastic. Then, immune cells (always used at a concentration of 1×10^5^ cells in 0.1 ml of cRPMI) isolated from spleens or tumor digests were added to the wells followed by the addition of CpG (1uM), rmIFNγ (1,000 U/ml) or both reagents. Plates were incubated at 37°C degrees and 5% CO2. 4T1 cell monolayers were examined under fluorescent and bright fields. Wells with 4T1-f cells in cRPMI alone were used as a reference for tumor cell growth. The assays were evaluated at 48h when totally confluent 4T1 monolayers were formed in the growth control wells. The addition of CpG, IFNγ or CpG plus IFNγ to 4T1-f cells did not alter the growth or survival of these cells out to 7 days in culture. To distinguish between cytostatic *vs* cytolytic effects, viable 4T1-f cells were evaluated at 48h, 72h and 7d after plating.

Immunofluorescence as well as brightfield images of wells displayed in Figures 5, 6, 7, 8 and Supplemental Figures 5-6 were taken at 100x magnification. To determine the percentage of killing of 4T1-f cells or inhibition of 4T1-f cell outgrowth, the percent of black areas (acellular spaces) of 6 non-overlapping high-power (400x) field equivalents was measured using public domain ImageJ software, and average percentage of killing ± SD was determined. Wells were scored by four independent evaluators and representative images are shown. Each treatment condition was furthermore set up in 2-3 microwells to verify technical reproducibility, and each condition was run from scratch at least twice to assess biologic reproducibility. Visualization of 4T1-f cells was performed using a Zeiss Axio Observer A1 microscope or the EVOS FL Auto Imaging System microscope. Similar assays were also run targeting the CT26 tGFP tumor cell line and 3T3 (tGFP or NIH Red) non-transformed mouse fibroblasts.

Similarly, to evaluate inducible tumoricidal properties in human monocytes, we employed the fluorescent (f) human breast cancer cell line MDA-MB-231-f as well as 4T1-f and 3T3-f as targets (each at 2×10^4^ cells in 0.1 ml cRPMI per well). Peripheral blood enriched human monocytes were obtained from healthy donors (as described previously) [72]. Briefly, countercurrent centrifugal elutriation was applied to leukapheresed peripheral blood. All isolated cells were myeloid (CD33+/CD13+) of which 90% were also CD14+. Isolated CD14+ and CD14- cells were ultrastructurally indistinguishable, indicating the CD33+CD14- cells were likely committed myeloid dendritic cells. Thawed cryopreserved monocytes were added to the wells (1×10^5^ cells in 0.1 ml cRPMI per well). To promote monocyte survival, rhGM-CSF (40 ng/ml) was added to the cultures at time 0. Exogenous rhIFNγ (25 ng/ml), TLR8 agonist resiquimod (3 μg/ml) (Invitrogen) and/or TLR4 agonist LPS (5 ng/ml) were also variably added to the cultures. Near confluence of MDA-MB-231-tGFP cells was observed at 96h in microwells receiving only GM-CSF and no monocytes. Evaluation of tumoricidal properties in human monocytes was assessed, following the same method used to evaluate tumoricidal properties in mouse cells co-cultured with 4T1-f cells.

### Clodronate liposome assays

To evaluate the role of phagocytic cells in the killing of 4T1-f tumor cells *in vitro*, clodronate (25 ng/microwell) or control liposomes (Encapsula Nano Sciences, Nashville TN) were variably added at time 0 to 96-well flat-bottom plates in which splenic Gr1dim cells exposed to CpG plus IFNγ were co-cultured with 4T1-f cells. Each condition was conducted in triplicate. Plates were incubated at 37°C degrees and 5% CO2. The effect of the clodronate or control liposomes was analyzed at 48h and 72h. The addition of the liposomes did not affect 4T1-f cell growth at the evaluated time points. The percentage of 4T1 cell killing was analyzed as previously explained (see above *in vitro* tumor killing assay).

### Evaluation of iNOS, serine protease and reactive oxygen species in 4T1 killing assays

To evaluate the mechanism involved in the killing of 4T1-f tumor cells by activated Gr1dim cells, inhibitors for iNOS (5mM L-NMMA, Sigma), serine proteases (4,000 U/ml trypsin inhibitor, Sigma) and reactive oxygen species (4,000 U/ml U catalase, Sigma) were variably added to wells in which splenic Gr1dim cells were exposed to CpG and/or CpG plus IFNγ and co-cultured with 4T1-f cells. Each condition was performed in duplicate, and 3 independent experiments were conducted. The effect of the inhibitors was initially analyzed at 48h when massive confluent monolayers were observed in the tumor growth control wells (4T1-f cells alone in cRPMI), and evaluated again after 7 days in culture. The addition of the inhibitors did not affect the growth of 4T1-f cells. The percentage of 4T1 cell killing was evaluated as previously explained (see above *in vitro* tumor killing assay).

### PD-L1 expression on 4T1 tumor cell line

4T1-f cells were plated in 96-well flat-bottom plates at a concentration of 2×10^4^ cells in 0.1 ml of cRPMI media. The plates were incubated for 20-60 min at 37°C degrees and 5% CO2 to allow 4T1-f cells to adhere to the plastic followed by the addition of CpG, IFNγ or CpG plus IFNγ (same concentrations used in 4T1-f killing assay, see above). 4T1 cells in medium alone were used as a control. Cells were incubated and PD-L1 expression on 4T1-f cells was evaluated after 48h in culture. To determine PD-L1 levels, 4T1 cells were detached by removing the media, incubating in PBS for 20 min at 37°C degrees and 5% CO2. Cells were detached by up and down pipetting. Cells were washed twice with FACs buffer and Fc Receptor blocking solution was added. Cells were stained with Brilliant violet 421 anti-mouse PD-L1 mAb (124315, Biolegend San Diego, CA) or isotype control. Cells were analyzed on a LSR Fortessa flow cytometer (BD Bioscience), using FACsDiva software (BD Biosicience).

### CSFE labeling

Enriched CD4 cells from spleens of naïve BALB/c mice, previously stained with Alexa Fluor 700 rat anti-mouse CD4 mAb (RMA-5 BD Bioscience), were washed, resuspended in PBS and labeled with CSFE (final working concentration 2.5 μM) (C34554, CellTrace CFSE Cell Proliferation Kit, Invitrogen). After 5 min incubation at room temperature, ice-cold cRPMI was added and cells were incubated on ice for 5 min. Then, cells were washed once and resuspended in fresh cRPMI (10 ml) at 37°C for 30 min, carefully mixing the cell suspension every 10 min (this step was done for a total of two times). Finally, cells were resuspended in fresh cRPMI and counted. Viability was assessed by trypan blue.

### CD4+ T-cell proliferation assay

Enriched CD4+ T-cells form spleens of naïve mice, previously labeled with CSFE and Alexa Fluor 700 rat anti-mouse CD4 mAb, were added at a concentration of 1.5×10^5^ cells/100 ul cRPMI per well to a 96-well round-bottom plate pre-coated with anti-CD3 and anti-CD28 mAbs (50 ul 2mg/ml each). Enriched myeloid Gr1dim or Gr1hi cells from spleens of untreated or CY+CpG-treated 4T1 TB mice, obtained by magnetic cell isolation (Myeloid-Derived Suppressor Cell Isolation Kit, Miltenyi Biotec), were variable added to the cultures at time 0 (1.5×10^5^ cells/100 ul cRPMI). Cells were incubated at 37°C for 72h. Flow cytometric analysis of T-cell proliferation was performed on gated CD4+ T-cells based on CFSE expression.

### Statistical analysis

All data are expressed as mean ± SD. ELISpot analyses were performed by using one-way ANOVA followed by Tukey's posttest for multiple comparisons. For therapy experiments in which binomial read out was appropriate (e.g., durable tumor rejection *vs* any other outcome), Fisher's exact test was employed. For comparison of means, unpaired Student's *t*-Test was used. All analyses were made using GraphPad Prism v5 software. Significance is shown as follows *p < 0.05, **p < 0.01, ***p < 0.001.

## SUPPLEMENTARY FIGURES AND TABLES



## Abbreviations

4T1-f: 4T1-fluorescent
CpG: CpG ODN 1826
CTL: cytolytic T-lymphocyte
CY: Cytoxan
i.p.: intraperitoneal
i.t.: intratumoral
KO: knock out
LN: lymph nodes
MDSCs: Myeloid-Derived Suppressor Cells
MTD: maximum tolerated dose
ODN: oligodeoxynucleotides
pIC: poly(I:C)
PD-1: Programmed death-1
PD-L1: Programmed death-ligand 1
s.c.: subcutaneous
TB: tumor-bearing
Teff: effector T-cells
TLRa: Toll-like receptor agonist (s)
Treg: regulatory T-cells
UF: unfractionated
WT: wild type
