# Supplementary material for: Definitive activation of endogenous antitumor immunity by repetitive cycles of cyclophosphamide with interspersed Toll-like receptor agonists
Source: Oncotarget. 2016 Jun 21;7(28):42919–42. doi: 10.18632/oncotarget.10190 (PMC5189997; doi:10.18632/oncotarget.10190)
Supplement: Supplementary file 1 [file oncotarget-07-42919-s001.pdf]

Definitive activation of endogenous antitumor immunity by repetitive cycles of cyclophosphamide with interspersed Toll-like receptor agonists

Supplementary Material

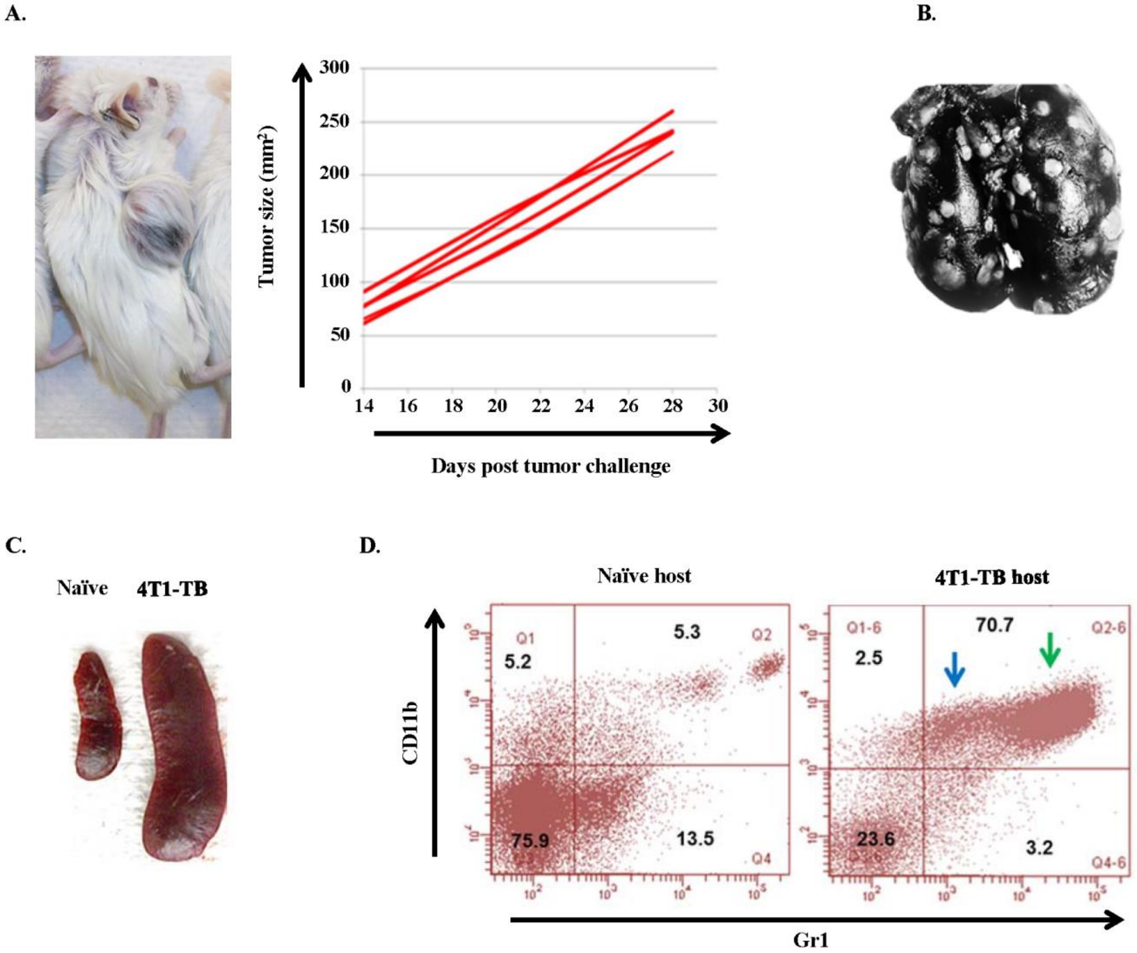

Supplemental Figure S1: 4T1 tumor is an aggressive and highly metastatic mouse breast tumor model. **A.** Tumor growth kinetics for ectopic 4T1 model, untreated control group. Untreated mice required sacrifice 4-5 weeks after tumor challenge ($1x10^6$ 4T1 cells) due to inanition or respiratory compromise from tumor progression. Each line represents a single mouse (n = 5). **B-D.** Hallmarks of 4T1 TB mice, week 4 post tumor challenge. **B.** Representative India ink-stained lungs from sacrificed untreated 4T1 TB mouse characterized by multiple metastatic nodules (white foci). **C.** Representative spleens from naïve mouse *vs* 4T1 TB mouse. **D.** Representative flow cytometry plots of total splenocytes from BALB/c naïve *vs* 4T1 TB mice stained with anti-CD11b and anti-Gr1 mAbs; percentage of cells is indicated. The arrows in the dot plot of the 4T1 TB host identify phenotypic characterization of MDSC subpopulations (blue arrow, monocytic Gr1dim; green arrow, granulocytic Gr1hi). Results shown in this figure are representative of over 10 biological replicates.

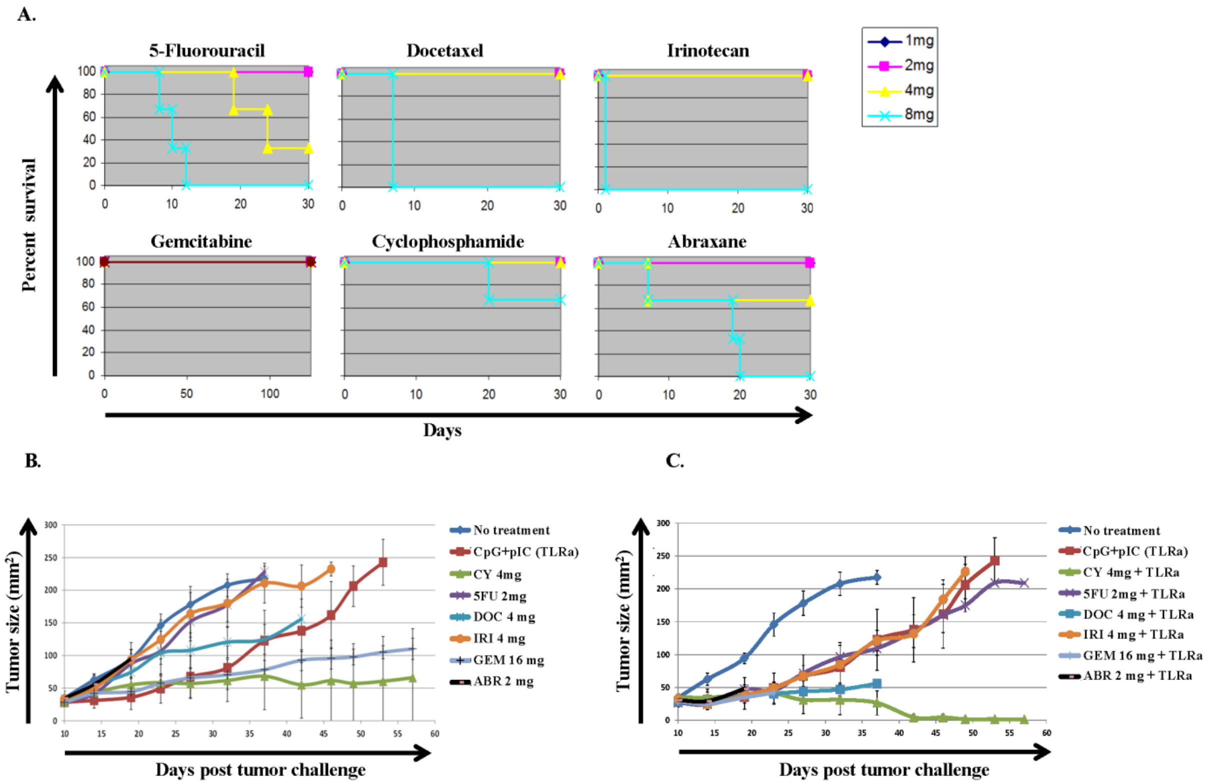

Supplemental Figure S2: Representative determinations of maximum tolerated doses (MTD), safety and efficacy of individual chemotherapy agents as monotherapy or in combination with TLRa (CpG+pIC). A. Individual commonly used chemotherapy agents were administered i.p. at the doses indicated to naïve non-TB BALB/c mice for a total of three doses on d0, d7 and d14 (n = 3 mice per group). MTD were defined as the highest weekly repetitive doses at which mice remained robust and did not require sacrifice for morbidities. Doses shown are for mice weighing 20 grams, receiving 5-fluorouracil (5FU), docetaxel (DOC), irinotecan (IRI), gemcitabine (GEM), cyclophosphamide (Cytoxan or CY), or nab-paclitaxel (Abraxane or ABR). Mice receiving 5-FU also received 1 mg leucovorin. Upper limit MTD was reached for all reagents except GEM. **B-C.** BALB/c mice were challenged with 4T1 cells ($1x10^6$) s.c., and then treated at d10 with weekly individual chemotherapeutic agents at MTD (d0 of each cycle), either (**B**) without TLRa or (**C**) with TLRa (i.p. CpG and intratumoral pIC, each at 100 µg on d0 and d3 of each cycle). TLRa alone treatment is also shown. Each line displays average tumor size ± SD of surviving mice for individual treatment groups (n = 4-6 mice) at designated time points. Each line terminates when all mice in the treatment group are no longer alive. At the end of experiment, mice in each treatment group were scored yes or no for durable regression, and analyzed in comparison to untreated TB mice by Fisher's exact test. Only combined CY+TLRa treatment was significantly different from untreated TB mice (**p = 0.0048). Parallel outcomes were observed in the CT26 and Panc02 tumor models (data not shown).

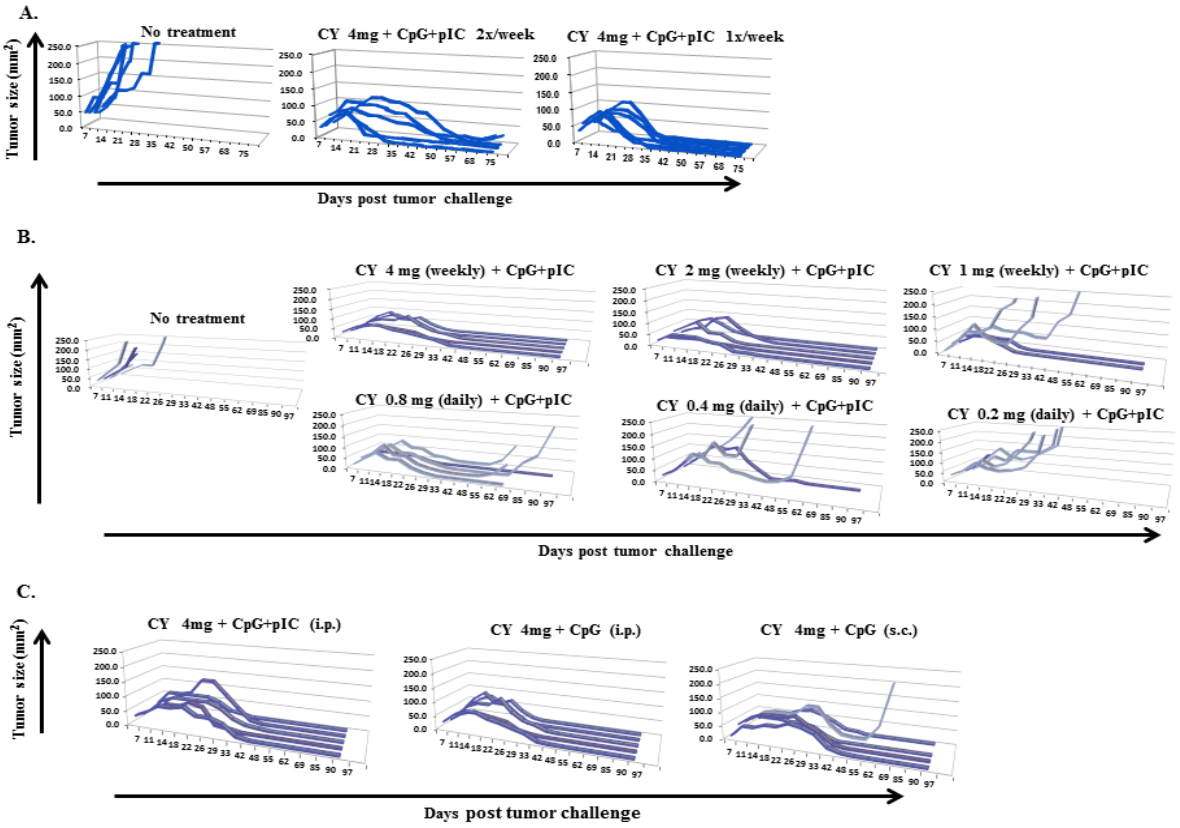

Supplemental Figure S3: Optimization of dose, route, and periodicity of CY plus TLRa therapy for tumor eradication. A-C. Plots of CT26 tumor growth *vs* days post tumor challenge, each line represents an individual mouse. **A.** Dosing TLRa only midcycle (d3) is therapeutically superior to TLRa given two times weekly, both early (d0) and midcycle (d3). CT26 TB mice were treated with CY (MTD 4 mg) once a week (d0 of each cycle) in combination with TLRa (CpG+pIC) both given i.p. either once (d3) or twice a week (d0 and d3). Using Fisher's exact test (scoring yes *vs* no for durable tumor regression), TLRa treatment confined to midcycle (d3) was significantly different from untreated TB mice, whereas combined early (d0) and midcycle (d3) TLRa dosing was not (**p = 0.0022 *vs* ns p = 0.1667). Parallel results were observed in the 4T1 model (data not shown). **B.** Comparison of decreasing doses of CY combined with single midcycle (d3) TLRa dosing (CpG+pIC) for weekly CY (top panel) *vs* daily CY (lower panel). Weekly doses of CY below 2 mg (100 mg/kg) or switching to daily CY rendered treatment less dependable. Only weekly CY at 4 mg or 2 mg was significantly different from untreated mice (**p = 0.0079 in both cases). Parallel results were observed in the 4T1 model (data not shown). **C.** In the CT26 tumor model, CY plus one TLRa is sufficient for sustained rejection, whether delivered s.c. or i.p. (same no treatment control group as in B). Weekly CY (MDT 4 mg) was given in conjunction with midcycle (d3) TLRa, comparing CpG alone *vs* CpG+pIC, and i.p. *vs* s.c. injections. Therapeutic outcomes for all tested TLRa variables were significantly different from no treatment (**p = 0.0079). Similar results were observed in the 4T1 model (data not shown).

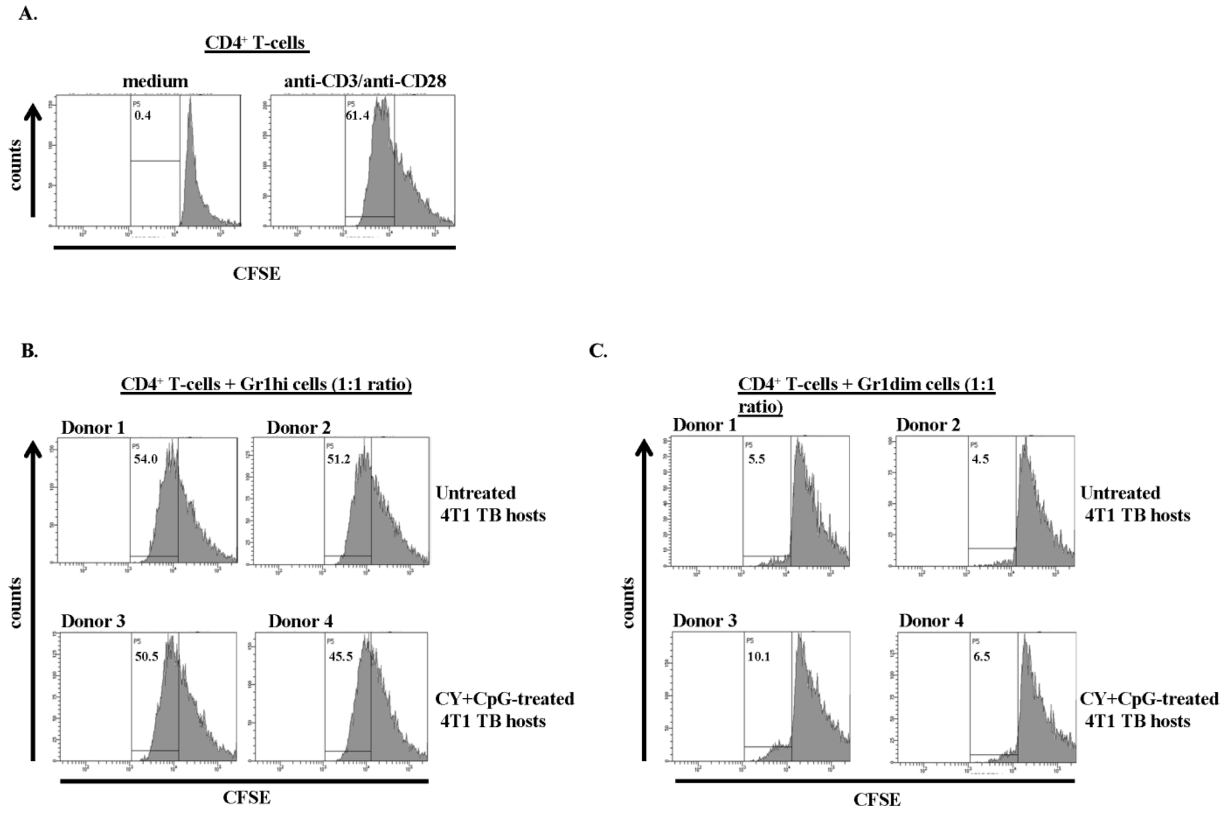

Supplemental Figure S4: Isolated myeloid Gr1dim cells from CY+CpG-treated 4T1 TB mice retain their capacity to inhibit T-cell proliferation. A-C. Isolated CD4+ T-cells ($1.5x10^5$) from naïve BALB/c mice were labeled with CFSE and anti-CD4 Alexa Fluor 700 mAb, cultured in the presence or absence of anti-CD3 and CD28 mAbs (**A**), and co-cultured or not with enriched CD11b+Gr1hi (**B**) or CD11b+Gr1dim cells (**C**) ($1.5x10^5$) from spleens of untreated or CY+CpG-treated mice. T-cell proliferation by CFSE dilution was measured after 72h culture. Numbers within the histogram plots indicate percentage of CD4+ T-cells which had proliferated. Data correspond to two biological replicates.

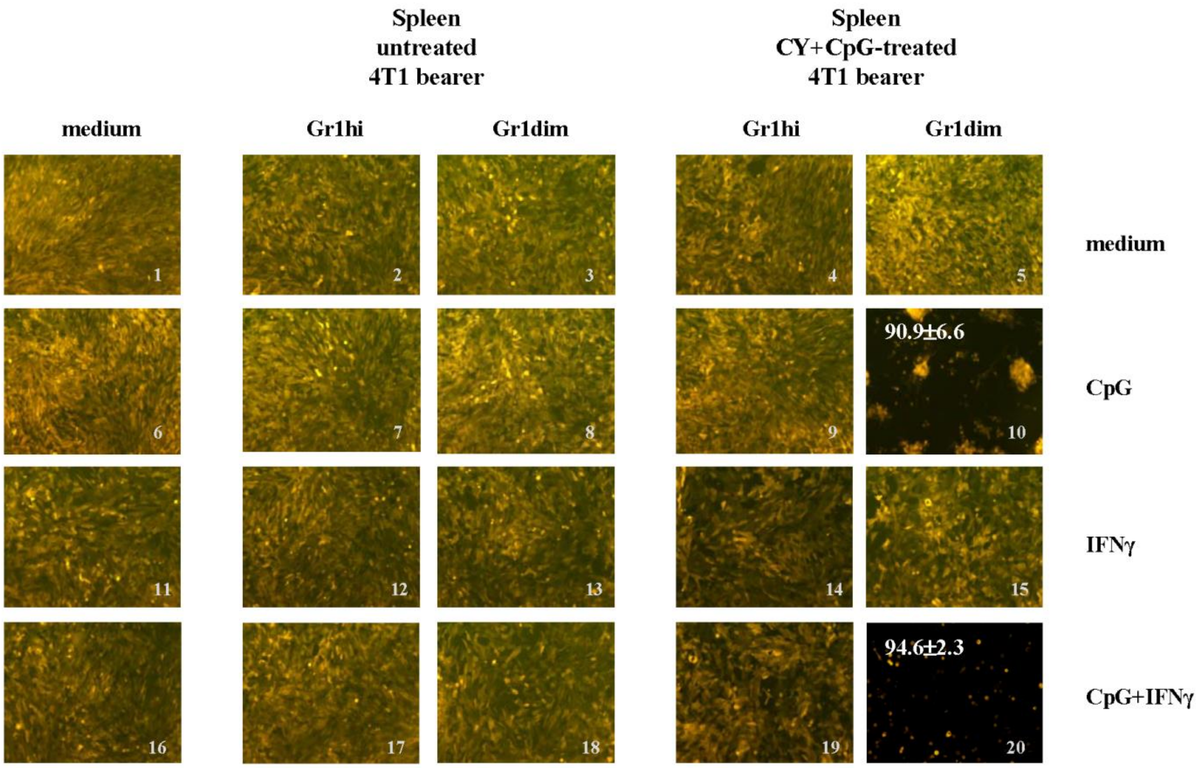

Supplemental Figure S5: **In contrast to untreated 4T1 TB mice, Gr1dim myeloid cells with tumoricidal properties are induced in CY+TLRa-treated mice.** Assessment of tumoricidal activity in Gr1 subsets (Gr1hi and Gr1dim) isolated from untreated *vs* CY+CpG-treated mice. Isolated splenic CD11b+Gr1+ subpopulations ($1x10^5$) were co-cultured with 4T1-f cells ($2x10^4$) in the presence or absence of CpG, IFNγ or CpG+IFNγ at time 0, and tumoricidal effect was evaluated from 48h to 7 days, and analyzed as in Figure 5. The percentage of tumor growth inhibition is denoted in white numbers (top left). Images without percentages represent confluent monolayers (killing below detection levels). Images were numerically labeled on the bottom right for the purpose of identification. Three untreated 4T1 TB mice and 10-15 CY+CpG-treated mice were pooled per experiment. Photos correspond to 48h after culture. Photos were taken using a Zeiss Axio Observer A1 microscope. Data are representative of 5 biological replicates. Statistical analysis presented in results was performed using Student's *t*-Test**.**

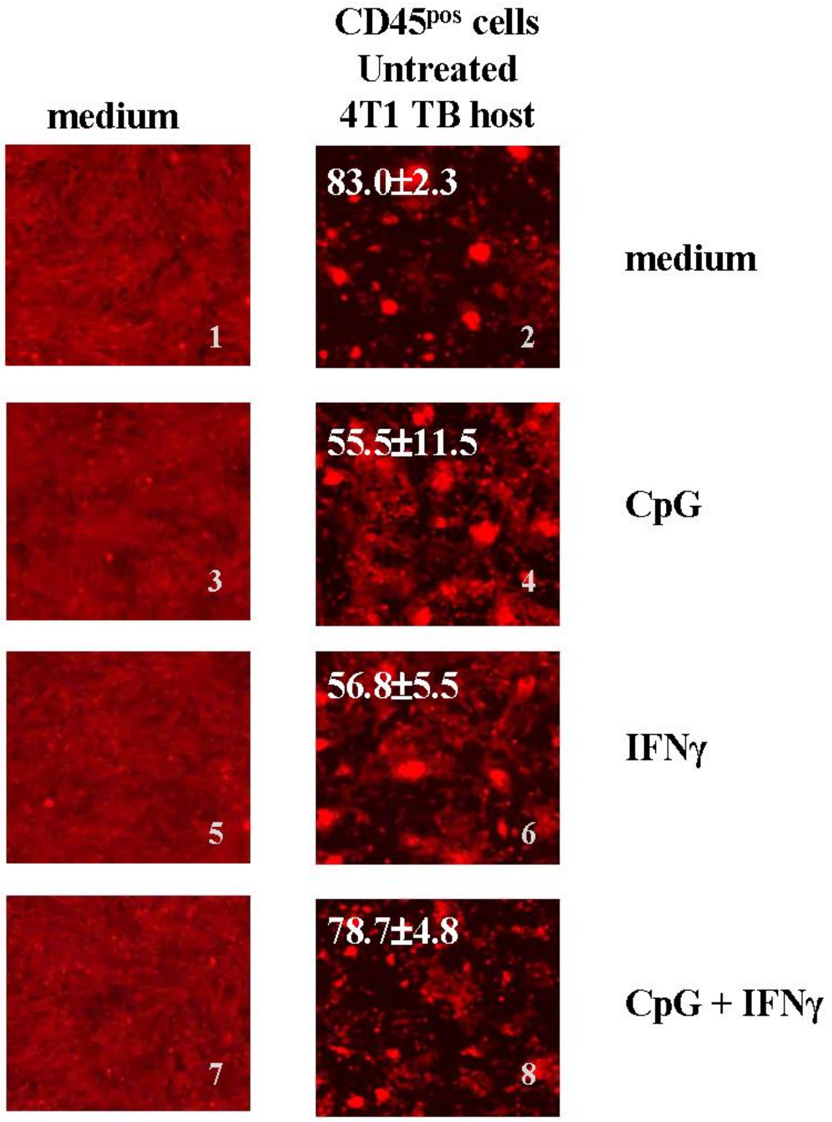

Supplemental Figure S6: Freshly isolated intratumoral leukocytes from untreated TB mice display tumoricidal properties *in vitro.* Isolated CD45^{pos} cells (1x10^{5}) from tumor digests of untreated 4T1 TB mice were co-cultured with 4T1-f cells (2x10^{4}) in the presence or absence of CpG, IFNγ or CpG+IFNγ at time 0. Tumoricidal activity was evaluated from 48h to 7 days as in Figure 5. The percentage of tumor growth inhibition is denoted in white numbers (top left). Images without percentages represent confluent monolayers (killing below detection levels). Images were numerically labeled on the bottom right for the purpose of identification. Statistical analysis presented in results was performed using Student's *t*-Test**.** Representative photos correspond to 48h in culture. Photos were taken using an EVOS FL Auto imaging system microscope. Data are representative of three biological replicates. Statistical analysis presented in results was performed using Student's *t*-Test**.**

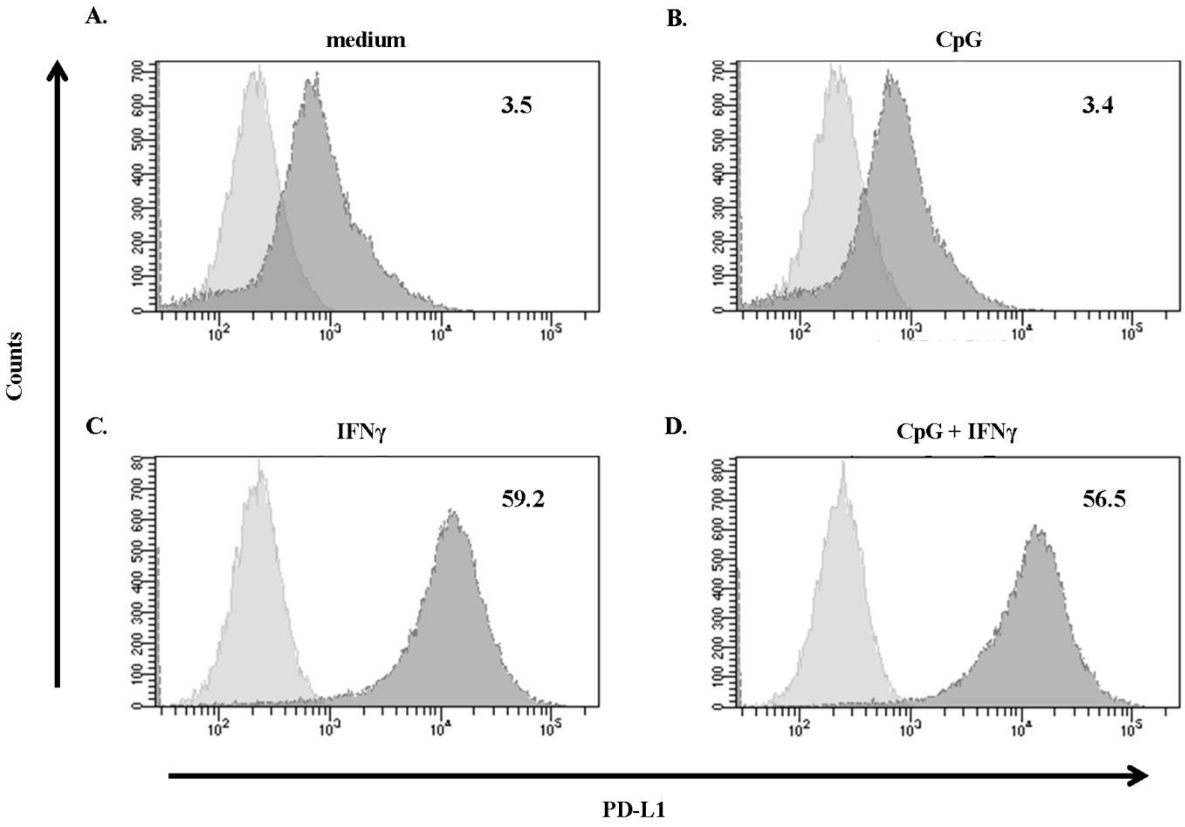

Supplemental Figure S7: Exposure to IFNγ up-regulates PD-L1 expression on 4T1 tumor cell line. A-D. Non-irradiated 4T1-f were cultured in the absence or presence of different stimulus; cRPMI alone (**A**), CpG (1 μM) (**B**), IFNγ (1000 U/ml) (**C**), or CpG+IFNγ (**D**), and PD-L1 expression was evaluated by flow cytometry after 48h in culture. Representative histogram plots of PD-L1 expression on 4T1-f cells. Lighter histogram corresponds to isotype control; darker histogram denotes PD-L1 expression. Numbers indicate MFI fold change with respect to isotype control.
